# CXCR2-Dependent Infiltration of Tumor-Associated Neutrophils Is Linked to Enhanced CD8^+^ T Cell Effector Function and Reduced Lung Metastasis in 4T1 Breast Cancer

**DOI:** 10.3390/ijms27073143

**Published:** 2026-03-30

**Authors:** Tiantian Li, Teizo Yoshimura, Miao Tian, Gakushi Nishida, Chunning Li, Masayoshi Fujisawa, Toshiaki Ohara, Akihiro Matsukawa

**Affiliations:** Department of Pathology and Experimental Medicine, Graduate School of Medicine, Dentistry and Pharmaceutical Sciences, Okayama University, Okayama 700-8558, Japan; p3ew3n25@s.okayama-u.ac.jp (T.L.); pd952tpz@s.okayama-u.ac.jp (M.T.); me428074@s.okayama-u.ac.jp (G.N.); 18640512230@163.com (C.L.); mfujisawa@okayama-u.ac.jp (M.F.); t_ohara@cc.okayama-u.ac.jp (T.O.)

**Keywords:** breast cancer, neutrophils, CD8^+^ T cells, chemokines, chemokine receptors, tumor microenvironment

## Abstract

Triple-negative breast cancer (TNBC) is characterized by prominent neutrophil infiltration; however, its significance remains controversial. Here, we investigated the role of neutrophil chemoattractant receptors in TNBC progression and metastasis. In contrast to *wild-type* (*WT*), *Fpr1*^−/−^, and *Fpr2*^−/−^ mice, neutrophils were almost completely absent in 4T1 tumors from *Cxcr2*^−/−^ mice, indicating a dominant role for CXCR2 in the recruitment of tumor-associated neutrophils, leading us to use *Cxcr2*^−/−^ mice for further studies. Primary tumor growth was comparable between *WT* and *Cxcr2*^−/−^ mice, whereas lung metastasis was significantly increased in *Cxcr2*^−/−^ mice, with reduced expression of inflammatory cytokines, chemokines and cytotoxic molecules, including granzyme B and perforin, in primary tumors and metastatic lungs of *Cxcr2*^−/−^ mice. In vitro, *WT*, but not *Cxcr2*^−/−^, neutrophils enhanced CD8^+^ T cell activation, partly via ICAM-1, and directly induced tumor cell death, supporting their anti-tumor function. To assess clinical relevance, transcriptomic data were analyzed. High neutrophil infiltration combined with elevated *CXCR2* expression, and to a lesser extent *CXCR1* expression, was associated with improved prognosis in patients with basal-like BC that largely overlaps with TNBC. Collectively, these findings suggest that CXCR2-mediated neutrophil recruitment exerts protective, anti-tumor effects and may represent a new prognostic marker for TNBC patients.

## 1. Introduction

The tumor microenvironment (TME) is increasingly recognized as a dynamic ecosystem composed of not only tumor cells but also components of the tumor stroma, including immune cells, blood vessels and extracellular matrix. These elements collectively influence tumor progression, angiogenesis, and metastatic dissemination. Among the diverse immune populations within the TME, neutrophils have recently gained attention because of their functional heterogeneity and context-dependent roles in cancer. There is increasing evidence that neutrophils play a crucial regulatory role in tumor initiation and progression. Additionally, tumor-associated neutrophils (TANs) can exert either pro- or anti-tumor activities, depending on the signals present in the TME [1]. Pro-tumor TANs, often overlapping with myeloid-derived suppressor cells (MDSCs), support tumor growth through immunosuppression and angiogenesis [2], whereas anti-tumor TANs exert cytotoxicity against tumor cells through the production of reactive oxygen (ROS), antibody-dependent cellular cytotoxicity (ADCC), degranulation of cytotoxic enzymes, and activation of CD8^+^ T cells [3,4]. Despite these insights, the precise contribution of neutrophils to tumor development and progression remains incompletely understood.

Trafficking of leukocytes into injured tissues is regulated by G-protein coupled receptors belonging to the chemokine receptor family and the classical chemoattractant receptor family [5]. CXCR1 and CXCR2 are chemokine receptors expressed on neutrophils that regulate their recruitment by recognizing the concentration gradients of ligands produced at injury sites. The role of CXCR2 has been well characterized in both humans and mice; however, the expression and functional relevance of CXCR1 in mice remains controversial, making its characterization in animal models challenging [6]. Formylpeptide receptors (FPRs), including FPR1 and FPR2, belong to the classical chemoattractant receptor family. Although originally identified as the receptors for N-formyl bacterial peptides, FPRs also bind to endogenous ligands, such as annexin A1 (ANXA1). ANXA1 primarily inhibits leukocyte migration and acts as a “stop signal”; however, certain ANXA1 fragments can instead promote leukocyte migration [7,8]. Cooperation between chemokine receptors and classical chemotactic receptors is critical for directing neutrophils to sites of tissue injury, including tumors [9]. However, the function of these receptor systems within the TME is not fully understood. In particular, it has yet to be fully elucidated whether FPR1/FPR2 also regulate the recruitment of TANs and contribute to tumor progression.

Breast cancer (BC) is a leading cause of cancer-related mortality among women worldwide, and disease progression and poor prognosis are strongly associated with increased infiltration of MDSCs [10]. Triple negative BC (TNBC) is a subtype of BC characterized by the absence of estrogen receptor (ER), progesterone receptor (PR), and human epidermal growth factor receptor 2 (HER2) expression. Patients with TNBC have a poorer prognosis compared to other BC subtypes [11]. Neutrophil recruitment into tumors is thought to depend largely on interactions between CXCR2 expressed on neutrophils and its ligands produced within the TME [12]. Human TNBC expresses higher levels of multiple CXCR2 ligands (CXCL1, 2, 3, 5, 6, 7 and 8) than luminal or HER2-positive tumors [13], suggesting that CXCR2-ligand interactions may drive neutrophil recruitment into TNBC. On the other hand, tumor necrosis is observed in 45–56% of TNBC cases [14], and ANXA1 is highly expressed in TNBC [7], suggesting that FPRs may also contribute to neutrophil recruitment in TNBC. Again, the role of FPRs in the trafficking of TANs and tumor progression in TNBC remains poorly defined.

CXCR2-deficient (*Cxcr2*^−/−^) mice have been used to study the role of TANs in the development of TNBC [15]. In the 4T1 model, systemic deletion of host CXCR2 did not affect primary tumor growth but reduced lung metastasis [16]. Intravenous injection of tumor cells derived from MMTV-PyMT tumors—a model for luminal AR subtype of TNBC [17]—into myeloid cell-specific *Cxcr2*^−/−^ mice also led to reduced lung colonization of the injected cells [18]. In contrast, when *Cxcr2*^−/−^ mice were crossed with MMTV-PyMT mice, CXCR2 deficiency led to increased primary tumor growth and enhanced lung metastasis [19]. In human TNBC cases, immunohistochemical analysis revealed that CXCR2-positive cells were mainly neutrophils, and higher CXCR2 expression was associated with a lower risk of relapse [13]. Thus, the impact of CXCR2-expressing neutrophils on TNBC progression remains unclear and additional studies are necessary to define the precise contribution of neutrophils to TNBC development and metastasis.

In the present study, we first evaluated the contribution of FPR1, FPR2, and CXCR2 to the recruitment of neutrophils in TNBC by orthotopically injecting 4T1 cells into *Fpr1*^−/−^, *Fpr2 *^−/−^, and *Cxcr2*^−/−^ mice. Contrary to our initial hypothesis, CXCR2 was identified as the dominant receptor regulating neutrophil infiltration into 4T1 tumors, leading us to use *Cxcr2*^−/−^ mice to evaluate the role of TANs in TNBC progression. Although the loss of neutrophil infiltration in *Cxcr2*^−/−^ mice had no effect on the primary tumor growth, it significantly increased lung metastasis. The expression of several immune-related genes was reduced in the TME of both primary tumors and lung metastases, likely resulting in increased colonization of cancer cells in the lung. In human basal-like BC cases that largely overlap with TNBC, high *CXCR2* or *CXCR1* expression in combination with neutrophil infiltration was associated with a more favorable prognosis. These results suggest that neutrophils infiltrating TNBC exert an anti-tumor activity and the expression of *CXCR2* and *CXCR1*, which, together with neutrophil infiltration, serve as prognostic markers for TNBC patients.

## 2. Results

### 2.1. Expression of FPR1, FPR2, and CXCR2 Ligands in 4T1 Cells and the 4T1 TME

**Figure 1:**
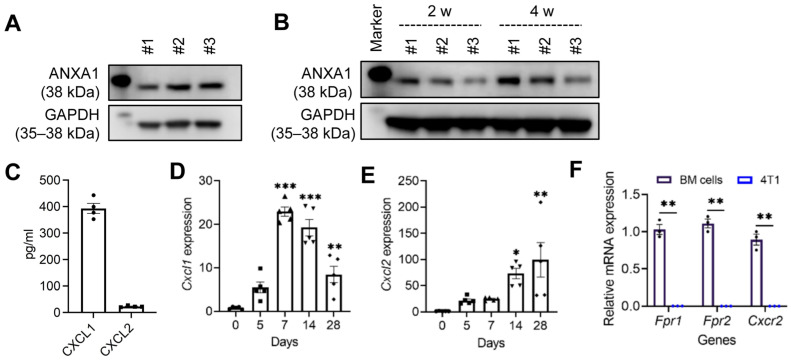
Expression of FPR1, FPR2, CXCR2, and their ligands in 4T1 cells and the TME. For in vitro experiments, 4T1 cells (1 × 10^4^) were cultured in 6-well plates for 2 days, after which, culture supernatants and cells were collected. For in vivo experiments, female mice were inoculated with 4T1 cells (1 × 10^5^ cells in 100 μL PBS) via mammary fat pad injection. At the indicated time points after injection, primary tumors were resected. (**A**,**B**) ANXA1 expression in 4T1 cells (**A**) and in the TME (**B**) was assessed by Western blotting using independent cell lysates (n = 3). (**C**) Concentrations of CXCL1 and CXCL2 in 4T1 culture supernatants were measured by ELISA (n = 4/group). (**D**,**E**) *Cxcl1* (**D**) and *Cxcl2* (**E**) mRNA expression in the 4T1 TME at different time points was analyzed by RT-qPCR (n = 4–5 mice/group). (**F**) *Fpr1*, *Fpr2*, and *Cxcr2* mRNA expression in 4T1 cells after 2 days of culture was examined by RT-qPCR. Data are presented as mean ± SEM. (**D**,**E**) Comparisons between day 0 and each time point were performed using unpaired Student’s *t*-tests. No symbol indicates that the difference was not significant. (**F**) Unpaired Student’s *t*-test. * *p* < 0.05, ** *p* < 0.01, *** *p* < 0.001.

Expression of the FPR1/2 ligand ANXA1 and the CXCR2 ligands CXCL1 and CXCL2 is detected in tumors of TNBC patients [20,21]. Therefore, we first examined whether ANXA1 and the CXCR2 ligands CXCL1 and CXCL2 are produced by 4T1 cells and within the 4T1 TME of WT BALB/c mice. The FPR1/FPR2 ligand ANXA1 was readily detectable in 4T1 cell lysates and in the 4T1 TME by Western blotting (Figure 1A,B). 4T1 cells also constitutively secreted the CXCR2 ligands CXCL1 and CXCL2 into the culture supernatants, with CXCL1 levels markedly higher than those of CXCL2 (Figure 1C). Neither *Cxcl1* nor *Cxcl2* expression was detected in mammary pads of normal WT BALB/c mice; however, the expression of both mRNAs was upregulated in the 4T1 TME. The level of *Cxcl1* gradually increased, peaked on day 7, and then declined (Figure 1D). The level of *Cxcl2* also gradually increased until day 28 (Figure 1E). By contrast, the expression of *Fpr1*, *Fpr2* or *Cxcr2* was not detected in 4T1 cells by RT-qPCR (Figure 1F). Collectively, these findings indicate that ligands for all three chemoattractant receptors were present in the 4T1 TME and may contribute to neutrophil recruitment into tumors.

### 2.2. CXCR2 as the Major Chemoattractant Receptor Regulating TAN Recruitment into 4T1 Tumors

**Figure 2:**
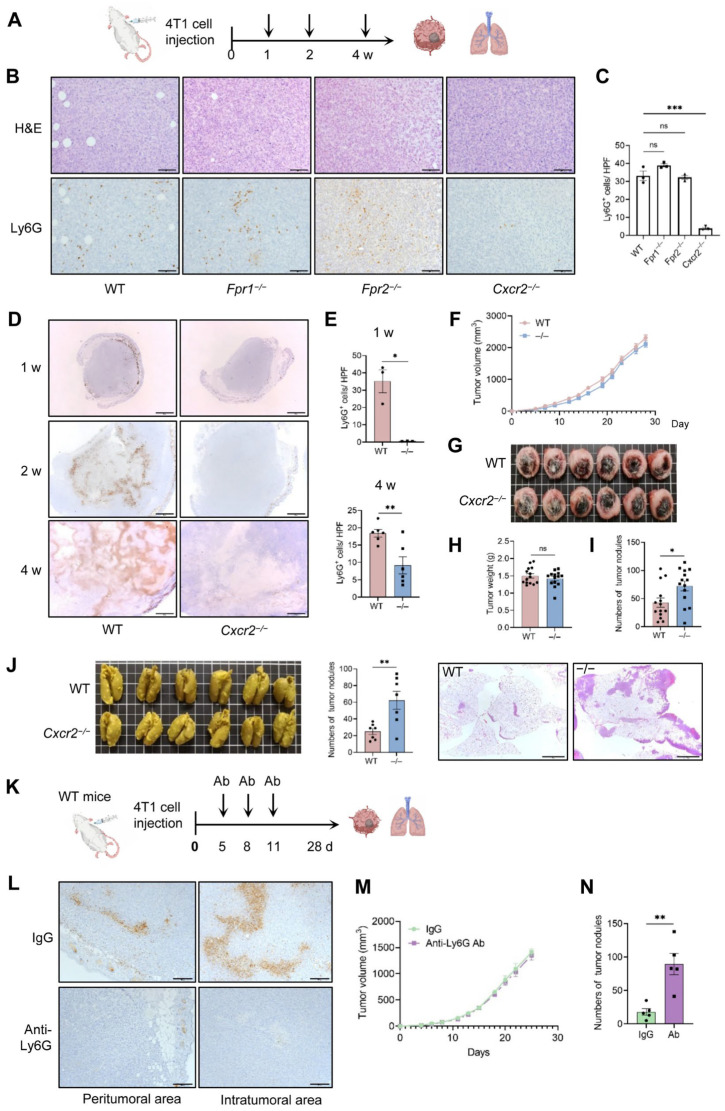
Host CXCR2 deficiency reduces neutrophil infiltration, does not affect primary tumor growth, but promotes lung metastasis. (**A**–**J**) 4T1 cells (1 × 10^5^) were inoculated into the mammary fat pad of female *WT*, *Fpr1*^−/−^, *Fpr2*^−/−^, and *Cxcr2*^−/−^ mice. Mice were euthanized 1, 2, and 4 weeks after injection, and tumors and lungs were harvested. (**A**) Diagram of the experimental design. (**B**) Representative H&E and anti-Ly6G immunohistochemical staining of primary tumor sections at 2 weeks. Scale bar = 100 μm. (**C**) Ly6G-positive cells were counted in non-necrotic tumor areas in WT, *Fpr1*^−/−^, *Fpr2*^−/−^, and *Cxcr2*^−/−^ mice. Ten random fields per section were analyzed at 200× magnification, and the average number of positive cells was used for comparison (n = 3 mice/group). (**D**) Representative anti-Ly6G immunohistochemical staining of primary tumors from WT or *Cxcr2*^−/−^ mice at the indicated time points. Scale bar = 1 mm. (**E**) Quantification of Ly6G^+^ cells in non-necrotic lesions at 1 (n = 3 mice/group) and 4 weeks (n = 6 mice/group) after injection, as described in (**C**). (**F**) Primary tumor size was measured three times per week (n = 14 mice/group). (**G**) Representative images of primary tumors at 4 weeks. (**H**,**I**) Primary tumors and spleen weights at 4 weeks (n = 14 mice/group). (**J**) Quantification of pulmonary metastasis (n = 7 mice/group) with representative images of Bouin’s-fixed lungs (left), metastatic nodule counts (middle), and H&E-stained sections (right; scale bar = 2 mm). (**K**–**N**) 4T1 cells (1 × 10^5^) were injected into the mammary pad of *WT* mice. Anti-Ly6G antibody (100 μg in 100 μL PBS) or control IgG (100 μg in 100 μL PBS) was intraperitoneally injected on days 5, 8, and 11. (**K**) Diagram of the experimental design. (**L**) Immunohistochemical staining of Ly6G^+^ neutrophil infiltration in primary tumors on day 14. Scale bar = 200 μm. (**M**) Primary tumor size was measured three times a week (n = 5 mice/group). (**N**) At 4 weeks, lungs were harvested and pulmonary metastases were quantified (n = 5 mice/group). Data are presented as mean ± SEM. (**C**) Comparisons between *WT* and each knockout mouse were performed using unpaired Student’s *t*-tests. (**E**,**H**–**J**,**N**) Unpaired Student’s *t*-tests. (**F**,**M**) Two-way ANOVA. * *p* < 0.05, ** *p* < 0.01, *** *p* < 0.001, ns; not significant.

To determine the contribution of FPR1, FPR2 and CXCR2 to neutrophil recruitment into 4T1 tumors, we orthotopically injected 4T1 cells into the mammary pad of *WT*, *Fpr1*^−/−^, *Fpr2*^−/−^, and *Cxcr2*^−/−^ mice and examined the infiltration of Ly6G-positive neutrophils into 4T1 tumors on day 14 by IHC (Figure 2A). Necrotic foci developed in tumors from mice of all 4 genotypes, and strong infiltration of Ly6G-positive cells was detected in and around the necrotic areas of *WT*, *Fpr1*^−/−^ and *Fpr2*^−/−^ mice. However, only a few Ly6G-positive cells were detected in tumors of *Cxcr2*^−/−^ mice (Figure S1). Infiltration of Ly6G-positive cells in non-necrotic areas was also markedly reduced in tumors from *Cxcr2*^−/−^ mice (Figure 2B,C). Neutrophil infiltration in tumors from *Cxcr2*^−/−^ mice was further examined at 1, 2, and 4 weeks after tumor cell inoculation. In *WT* mice, neutrophil infiltration was detected in the peripheral area surrounding tumors at 1 week and predominantly in and around the necrotic foci at 2 and 4 weeks (Figure 2D). By contrast, neutrophil infiltration was not evident at 1 week in *Cxcr2*^−/−^ mice and remained significantly reduced at 4 weeks (Figure 2E). Collectively, these results demonstrate that CXCR2 is the key receptor regulating the recruitment of TANs into 4T1 tumors, prompting us to further investigate the role of TANs in 4T1 tumor progression using *Cxcr2*^−/−^ mice.

### 2.3. Host CXCR2 Deficiency Does Not Affect Primary Tumor Growth but Increases Lung Metastasis

We next examined whether reduced TAN infiltration due to host CXCR2 deficiency affects primary tumor growth and pulmonary metastasis. No significant differences were observed in tumor volume, tumor weight and spleen weight between the two groups (Figure 2F–I). However, the number of lung metastatic nodules was markedly increased in *Cxcr2*^−/−^ mice (Figure 2J). No detectable macrometastases were observed in other organs, including the liver, spleen, brain, and bone, upon gross examination in both *WT* and *Cxcr2*^−/−^ mice. To obtain evidence that reduced TAN infiltration was responsible for increased lung metastasis in *Cxcr2*^−/−^ mice, we depleted neutrophils in *WT* mice by intraperitoneally administering anti-Ly6G antibody on days 5, 8 and 11 after 4T1 cell inoculation (Figure 2K). This treatment effectively reduced the number of Ly6G-positive neutrophils in primary tumors (Figure 2L) without affecting primary tumor growth (Figure 2M). Notably, neutrophil depletion significantly increased the number of lung metastases (Figure 2N). These findings support our view that impaired TAN infiltration accelerates metastatic progression without substantially impacting primary tumor growth in this model.

### 2.4. Impaired TAN Infiltration Compromises Anti-Tumor Immune Responses in the 4T1 TME

To investigate the mechanisms through which reduced TAN infiltration in the TME results in enhanced lung metastasis, we assessed tumor vascularization. Angiogenesis in the primary tumor is a critical process in cancer cell metastasis [22,23]. CD31^+^ microvessel density did not differ between *WT* and *Cxcr2*^−/−^ tumors (Figure S2A). Similarly, ERG^+^ endothelial cell numbers were comparable on day 14, with only a slight increase in *Cxcr2*^−/−^ tumors on day 28 (Figure S2B). VEGF-A levels were also similar between the two groups (Figure S2C). These data indicate that reduced TAN infiltration does not substantially alter tumor angiogenesis in 4T1 primary tumors.

Neutrophils produce and release a wide array of proinflammatory cytokines and chemokines that recruit and activate other immune cells [24], including macrophages [25] and lymphocytes [26], thereby shaping the initiation and resolution of immune responses.

**Figure 3:**
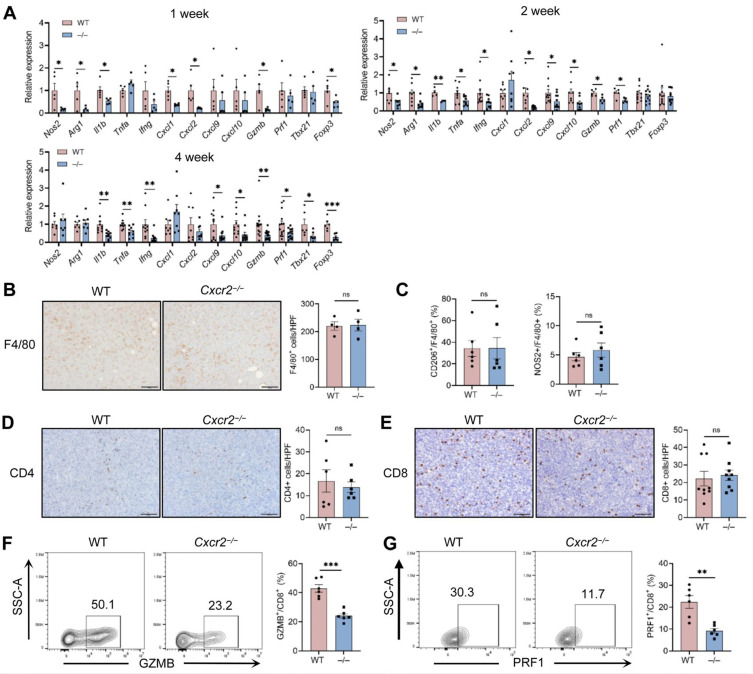
Host CXCR2 deficiency reduces inflammatory mediators without affecting macrophage or T cell infiltration. 4T1 cells (1 × 10^5^) were inoculated into the mammary fat pad of female WT and *Cxcr2*^−/−^ mice. Mice were euthanized at the indicated time points after injection, and primary tumors were harvested. (**A**) Total RNA was isolated from tumors at 1, 2 and 4 weeks, and mRNA expression levels were analyzed by RT-qPCR (n = 5–12 mice/group). (**B**) Left: Representative immunohistochemical staining of F4/80^+^ macrophages at 4 weeks. Scale bar = 100 μm. Right: The number of F4/80^+^ macrophages at 4 weeks were quantified by randomly selecting 10 fields per section under 200× magnification, and the average count was used for comparison (n = 4 mice/group) (**C**) The frequency of CD206^+^ or NOS2^+^ cells among F4/80^+^ macrophages in tumors from *WT* and *Cxcr2*^−/−^ mice at 4 weeks was analyzed by flow cytometry (n = 6 mice/group). (**D**,**E**) Left: Representative immunohistochemical staining of CD4^+^ (**D**) and CD8^+^ (**E**) cells at 4 weeks. Scale bar = 100 μm. Right: The number of CD4^+^ (**D**) or CD8^+^ (**E**) positive T cells at 4 weeks was quantified by randomly selecting 10 fields per section under 200× magnification, and the average count was used for comparison (n = 6–9 mice/group). (**F**,**G**) The frequency of GZMB^+^ (**G**) or PRF1^+^ (**F**) cells among tumor-infiltrating CD8^+^ T cells from WT and *Cxcr2*^−/−^ mice (2 weeks after tumor inoculation) was analyzed by flow cytometry (n = 6 mice/group). Data are presented as mean ± SEM. (**A**) Comparisons between *WT* and *Cxcr2*^−/−^ groups for each gene were performed using unpaired Student’s *t*-tests. No symbol indicates that the difference not significant. (**B**–**G**) Unpaired Student’s *t*-tests. * *p* < 0.05, ** *p* < 0.01, *** *p* < 0.001, ns; not significant.

To determine how reduced TAN infiltration affects the tumor immune landscape, we examined the expression of several immune-related genes (Figure 3A). *Nitrogen oxide synthase 2 (Nos2)*, *arginase 1 (Arg1)* and *Cxcl2* expression levels were reduced in tumors of *Cxcr2*^−/−^ mice at 1 and 2 weeks but not at 4 weeks. *Tumor necrosis factor-α (Tnfa)* and *interferon-γ (Ifng)* expression was not different between *WT* and *Cxcr2*^−/−^ tumors but the expression of both mRNAs declined at 2 and 4 weeks, while *interleukin-1β (Il1b)* expression was consistently lower in *Cxcr2*^−/−^ tumors. Together, these findings suggest that reduced TAN infiltration is associated with a dampened inflammatory state within the TME. Gene-expression profiling also revealed significant reductions in the expression of key T cell effector molecules, including *granzyme B (Gzmb)*, *perforin 1 (Prf1)*, *T-box transcription factor 21 (Tbx21)*, and *forkhead box P3 (Foxp3)* in *Cxcr2*^−/−^ tumors at all time points. The expression of the CD8^+^ T cell chemoattractants *Cxcl9* and *Cxcl10* was reduced in *Cxcr2*^−/−^ tumors at 2 and 4 weeks (Figure 3A).

We next examined the infiltration of macrophages and T cells at 4 weeks after 4T1 inoculation by IHC and flow cytometry. IHC analyses showed no significant differences in the density of F4/80^+^ macrophages (Figure 3B). According to the flow cytometry analysis, there was no significant difference in the proportions of NOS2^+^ (M1) or CD206^+^ (M2) macrophages among F4/80^+^ macrophages between *WT* and *Cxcr2*^−/−^ tumors (Figure 3C). Although the expression of *Cxcl9* and *Cxcl10* was reduced in *Cxcr2*^−/−^ tumors, IHC analysis revealed no significant difference in the number of tumor-infiltrating CD4^+^ and CD8^+^ T cells between the groups (Figure 3D,E), suggesting the contribution of alternative chemokines, such as CCL3, CCL4 and CCL5 [27], to the recruitment of CD4^+^ and CD8^+^ T cells.

As demonstrated above, reduced TAN infiltration in *Cxcr2*^−/−^ tumors was associated with downregulated expression of immune-related genes but not with CD8^+^ T cell infiltration. These findings led us to speculate that the absence of neutrophils may impair the cytotoxic function of CD8^+^ T cells. Therefore, we examined the expression of the cytotoxic effector molecules GZMB and PRF1 by tumor-infiltrating CD8^+^ T cells using flow cytometry. The frequencies of both GZMB^+^ and PRF1^+^ among CD8^+^ T cells were significantly decreased in *Cxcr2*^−/−^ tumors compared with *WT* controls (Figure 3F,G). These findings suggest that reduced TAN infiltration does not substantially influence the magnitude of macrophage recruitment or polarization, nor overall T cell recruitment within primary tumors, but reduces inflammatory responses and CD8^+^ T cell effector function. This observation contrasts with the well-recognized immunosuppressive roles attributed to neutrophils in other tumor models [26]. Notably, 4T1 tumors are highly aggressive and largely refractory to immunotherapy [28], which may explain why the observed alterations in the immune microenvironment were insufficient to affect the growth of 4T1 primary tumors.

### 2.5. CXCR2 Deficiency Limits Neutrophil Accumulation in Metastatic Lung Tumors and Impairs the Development of Metastatic Niche

**Figure 4:**
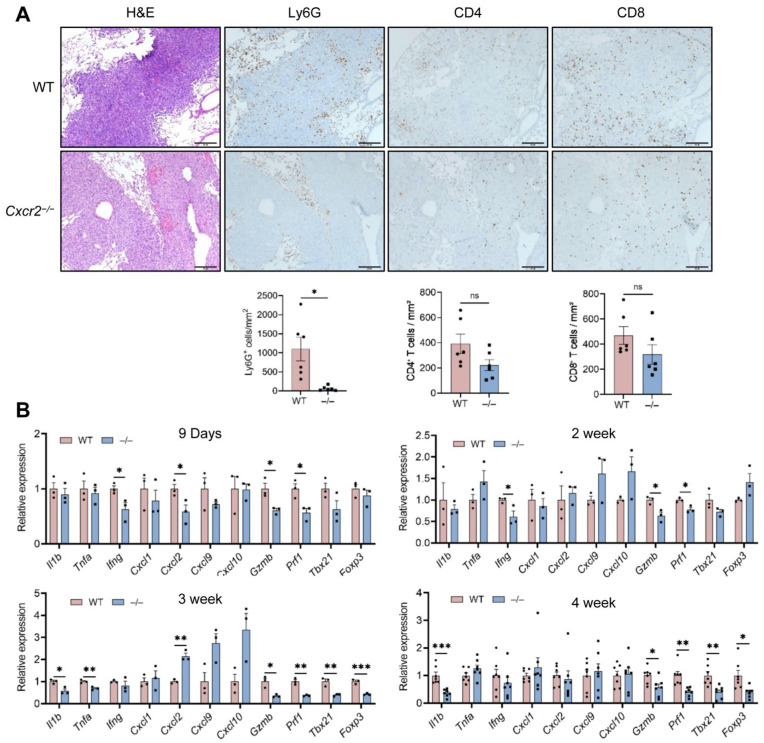
CXCR2 deficiency reduces neutrophil infiltration and immune-related gene expression in lung metastases. 4T1 cells (1 × 10^5^) were inoculated into the mammary fat pad of female WT and *Cxcr2*^−/−^ mice. Mice were euthanized at the indicated time points after injection, and lungs were harvested. (**A**) Infiltration of Ly6G^+^, CD4^+^ or CD8^+^ T cells in metastatic nodules at 4 weeks was examined by immunohistochemistry using anti-Ly6G, anti-CD4 or anti-CD8 antibodies. Upper panels: Representative H&E and immunohistochemical staining images. Scale bar = 200 μm. Lower panels: Ly6G^+^, CD4^+^ and CD8^+^ cells within metastatic lesions were counted, and cell density was calculated as the total number of positive cells divided by the total metastatic lesion area per mouse (cells/mm^2^). Each data point represents one mouse (n = 6 mice/group). (**B**) Lungs were harvested from tumor-bearing *WT* or *Cxcr2*^−/−^ mice at 9 days, and 2, 3 and 4 weeks after inoculation. Total RNA was extracted and gene expression levels were evaluated by RT-qPCR (n = 3–7 mice/group). Data are presented as mean ± SEM. (**A**) Unpaired Student’s *t*-test. (**B**) Comparisons between *WT* and *Cxcr2*^−/−^ groups for each gene were performed using unpaired Student’s *t*-tests. No symbol indicates that the difference was not significant. * *p*  < 0.05, ** *p*  <  0.01, *** *p*  <  0.001. ns; not significant.

Metastatic progression depends on the cross-talk between tumor cells and receptive target tissues [29]. We therefore investigated whether CXCR2 deficiency impairs TAN recruitment to metastatic lung tumors. Mice were systemically perfused with PBS, and the lungs were lavaged to more accurately assess neutrophils associated with metastatic nodules. In lung metastatic nodules in *Cxcr2*^−/−^ mice, accumulation of Ly6G^+^ cells, but not CD4^+^ or CD8^+^ T cells, was markedly reduced (Figure 4A).

A recent single-cell RNA sequencing analysis of the developing lung metastatic niche after 4T1 cell inoculation [30] revealed a temporal shift in immune composition. The pre-metastatic and early metastatic niches were composed of immune cells with an anti-tumor phenotype, but the metastatic niche at a later stage displayed a pro-tumor phenotype. This transition was closely associated with dynamic changes in neutrophil and monocyte behavior over time. We analyzed the expression of selected cytokines, chemokines and other immune-related genes in whole lung tissues at different time points following tumor cell inoculation. Nine days after the inoculation, the expression of *Ifng* was significantly reduced in *Cxcr2*^−/−^ mice compared with *WT* controls. This reduction persisted at 2 weeks but was no longer evident at 3 or 4 weeks. In contrast, expression of the cytotoxic effector molecules *Gzmb* and *Prf1* was consistently decreased in *Cxcr2*^−/−^ mice at all examined time points (Figure 4B). These findings strongly suggest that CXCR2-dependent TAN infiltration is critical for augmenting and maintaining CD8^+^ T cell cytotoxic capacity in the metastatic lung, and that the increased metastatic burden observed in *Cxcr2*^−/−^ mice likely reflects the loss of neutrophil-mediated promotion of CD8^+^ T cell effector function.

### 2.6. Neutrophils Exhibit Both Direct and Indirect Cytotoxic Activities Against Cancer Cells

**Figure 5:**
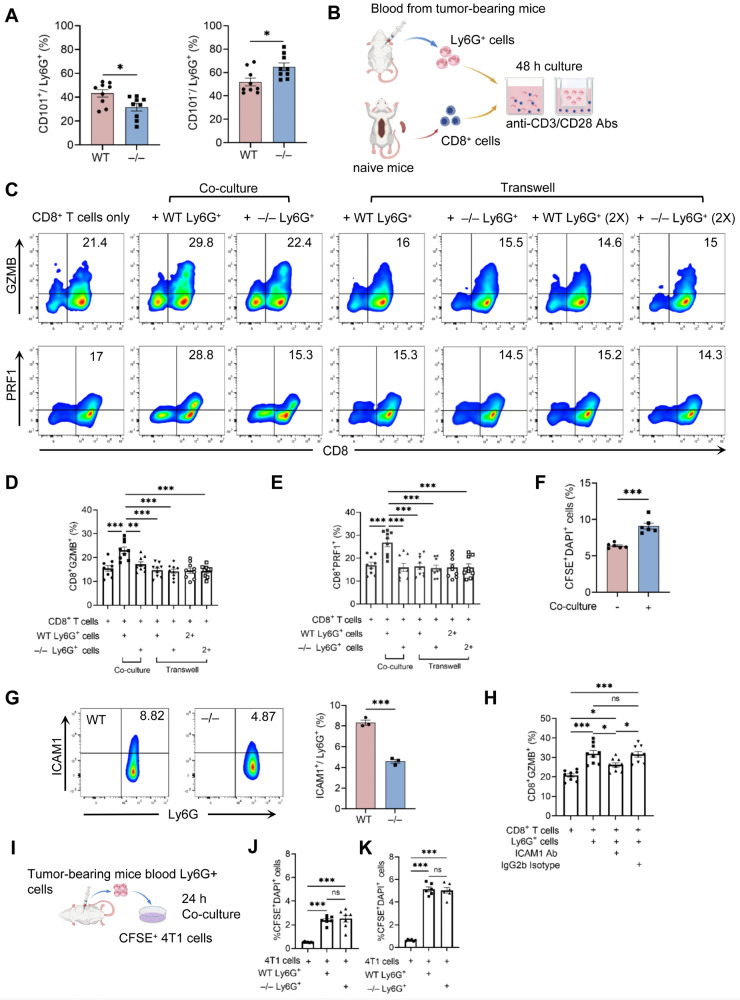
CXCR2 deficiency impairs neutrophil-driven CD8^+^ T cell cytotoxicity. (**A**) Flow cytometric analysis of peripheral blood neutrophils from tumor-bearing *WT* or *Cxcr2*^−/−^ mice (n = 9 mice/group). The frequencies of CD101^+^ cells (left) and CD101^−^ cells (right) among Ly6G^+^ cells are shown. (**B**–**E**) CD8^+^ T cells (1 × 10^6^) isolated from naïve WT mice were co-cultured with neutrophils (1 × 10^6^) from tumor-bearing *WT* or *Cxcr2*^−/−^ mice in 24-well tissue culture plates coated with anti-CD3/CD28 antibodies or using cell culture inserts. After 48 h, cells were collected and intracellular GZMB or PRF1 expression in CD8+ T cells was analyzed by flow cytometry. (**B**) Diagram of the experimental design. (**C**) Representative flow cytometry plots showing the expression of GZMB and PRF1 in CD8+ T cells. (**D**,**E**) Frequencies of CD8^+^GZMB^+^ (**D**) and CD8^+^PRF1^+^ (**E**) (n = 9 mice/group). (**F**) Stimulated CD8^+^ T cells (alone or co-cultured with *WT* neutrophils) were added to CFSE-labeled 4T1 cells (effector-to-target ratio of 10:1) for 48 h. Tumor cell death (CFSE^+^DAPI^+^) was assessed by flow cytometry (n = 6 mice/group). (**G**) Surface ICAM-1 expression on peripheral blood neutrophils from tumor-bearing *WT* and *Cxcr2*^−/−^ mice was analyzed by flow cytometry (n = 3 mice/group). (**H**) CD8^+^ T cells and neutrophils were co-cultured at a 1:1 ratio with an anti-ICAM-1 blocking antibody (1 μg/mL) or the corresponding isotype control. GZMB expression in CD8^+^ T cells was assessed by flow cytometry (n = 9 mice/group). (**I**–**K**) CFSE-labeled 4T1 cells were co-cultured with neutrophils at a 10:1 ratio in RPMI-1640 supplemented with 10% or 0.5% FBS for 24 h. (**I**) Diagram of the experimental design. (**J**,**K**) Tumor cell death (CFSE^+^DAPI^+^) was quantified under 10% FBS conditions (**J**); n = 7 mice/group) and 0.5% FBS conditions ((**K**); n = 6 mice/group). Data are presented as mean ± SEM. (**A**,**F**,**G**) Unpaired Student’s *t*-tests. (**D**,**E**,**H**,**J**,**K**) One-way ANOVA. No symbol indicates that the difference was not significant. * *p* < 0.05, ** *p* < 0.01, *** *p* < 0.001, ns; not significant.

To examine whether neutrophils have the capacity to promote CD8^+^ T cell activation, we performed co-culture experiments in vitro. In the 4T1 model, tumor cells produce growth factors, such as granulocyte-macrophage colony-stimulating factor (GM-CSF) and granulocyte colony-stimulating factor (G-CSF), which promote extramedullary granulopoiesis and expansion of low-density neutrophils containing immature subsets [31]. We analyzed the proportion of immature (CD101^−^) and mature (CD101^+^) neutrophils among Ly6G^+^ cells in our preparations using flow cytometry. As expected, tumor-bearing *WT* and *Cxcr2*^−/−^ mice exhibited a substantial increase in CD101^−^ immature neutrophils, with a further increase observed in *Cxcr2*^−/−^ mice (Figure 5A). These neutrophils were co-cultured with CD8^+^ T cells isolated from naïve *WT* mice in the presence of anti-CD3 and anti-CD28 Abs (Figure 5B). After 48 h, co-culture with *WT* neutrophils significantly increased the frequencies of GZMB^+^ and PRF1^+^ CD8^+^ T cells (Figure 5C–E). By contrast, neutrophils from *Cxcr2*^−/−^ mice failed to increase the frequencies of GZMB^+^ or PRF1^+^ CD8^+^ T cells (Figure 5C–E). Using a transwell system, we assessed whether direct cell–cell contact was required. When CD8^+^ T cells and neutrophils were separated by a membrane, WT neutrophils failed to increase the frequencies of GZMB^+^ or PRF1^+^ CD8^+^ T cells (Figure 5C–E), indicating that contact-dependent mechanisms are essential for this effect. To evaluate the cytotoxic function, splenic *WT* CD8^+^ T cells were first co-cultured with WT neutrophils for 48 h and then incubated with CFSE-labeled 4T1 cells for an additional 4 h. CD8^+^ T cells previously co-cultured with neutrophils showed enhanced cytotoxic activity compared with controls (Figure 5F).

We next explored potential mechanisms through which neutrophils enhance CD8^+^ T cell cytotoxic function. Intracellular adhesion molecule 1 (ICAM-1), originally characterized on endothelial cells as a key factor of leukocyte adhesion, is also expressed on immune cells and contributes to immune synapse formation and supports T cell activation [32]. 

ICAM-1 is also expressed on neutrophils and inhibition of ICAM-1 significantly reduces T cell activation [33], suggesting a potential role for neutrophils in promoting anti-tumor T cell activation through direct interaction via ICAM-1. Flow cytometric analysis revealed that approximately 8% of circulating neutrophils from tumor-bearing WT mice were ICAM-1^+^ compared with ~4% in tumor-bearing Cxcr2^−/−^ mice (Figure 5G). To evaluate the functional role of ICAM-1, we added a blocking antibody against ICAM-1 during neutrophil–CD8^+^ T cell co-culture and evaluated the frequency of GZMB^+^ CD8^+^ T cells. ICAM-1 blockade partially, but significantly, reduced the frequency of GZMB^+^ CD8^+^ T cells (Figure 5H). These findings suggest that *WT* neutrophils can indeed promote CD8^+^ T cell activation through an ICAM-1-dependent mechanism, with other mechanisms likely involved.

Neutrophils can also exert direct cytotoxicity against tumor cells [34], raising the possibility that qualitative defects may contribute to the enhanced metastasis observed in *Cxcr2*^−/−^ mice. To address this, we co-cultured CFSE-labeled 4T1 cells with Ly6G^+^ peripheral blood neutrophils from tumor-bearing *WT* or *Cxcr2*^−/−^ mice for 24 h under standard culture conditions (10% FBS). Tumor cell death was ~0.5% in the absence of neutrophils and increased to ~2.3% in the presence of either *WT* and *Cxcr2*^−/−^ neutrophils (Figure 5J). Because serum contains antioxidant factors, including albumin, which may suppress neutrophil effector functions [35], we reduced the FBS concentration to 0.5%, which increased tumor cell death to ~5.5% (Figure 5K). However, no significant difference in cytotoxicity was observed between *WT* and *Cxcr2*^−/−^ neutrophils under either condition (Figure 5J,K). Taken together, our findings suggest that the increased lung metastasis observed in *Cxcr2*^−/−^ mice is largely attributable to a decreased number of neutrophils capable of promoting CD8^+^ T cell activation and direct tumor cell killing.

### 2.7. Host CXCR2 Deficiency Promotes Tumor Growth and Lung Metastasis in the CT26 Colon Cancer Model

**Figure 6:**
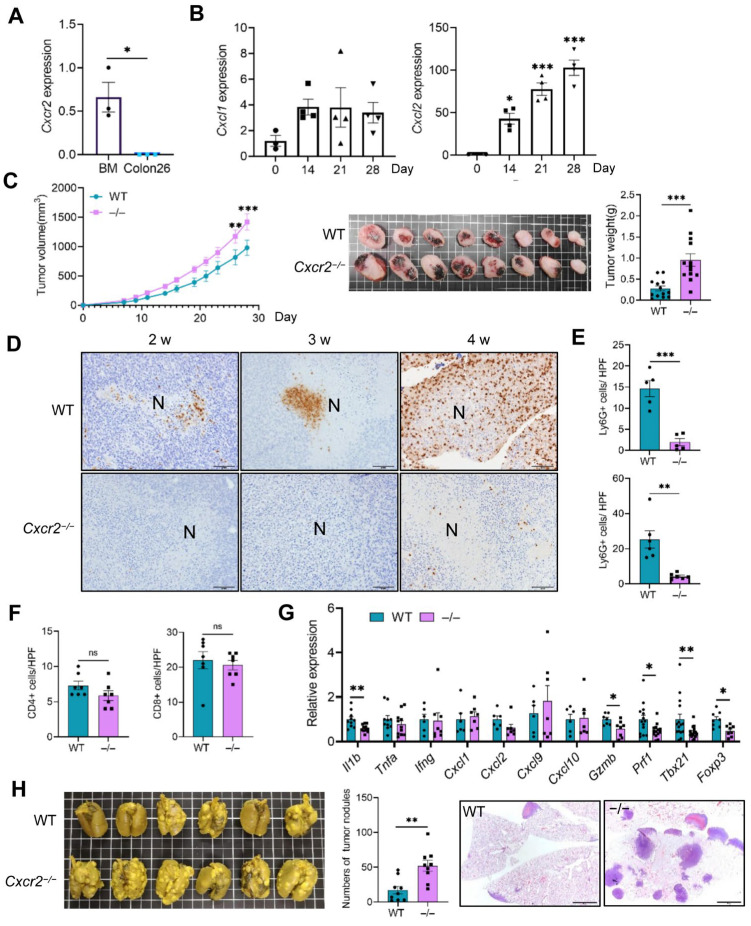
Disruption of CXCR2-dependent neutrophil recruitment promotes lung metastasis in the CT26 murine colon cancer model. (**A**) *Cxcr2* mRNA expression in CT26 cells was analyzed by RT-qPCR; bone marrow (BM) cells served as a positive control (n = 3/group). (**B**–**G**) CT26 cells (1 × 10^5^) were subcutaneously injected into the dorsal flank of *WT* and *Cxcr2*^−/−^ mice. Mice were euthanized at the indicated time points after injection, and primary tumors and lungs were harvested. (**B**) RT-qPCR analysis of *Cxcl1* and *Cxcl2* mRNA expression in CT26 tumors at different time points (n = 3–4 mice/group). (**C**) (Left) Tumor size was measured three times per week. After 4 weeks, tumors were harvested (middle) and weighed (Right) (n = 13 mice/group). (**D**) Representative anti-Ly6G immunohistochemical staining of primary tumor sections at 2, 3, and 4 weeks. Scale bar = 100 μm. (**E**) Ly6G-positive neutrophils were counted in non-necrotic areas of WT and *Cxcr2*^−/−^ tumors. The number of positive cells at 2 and 4 weeks was quantified by randomly selecting 10 fields per section at 200× magnification, and the average count was used for comparison (n = 5–6 mice/group). (**F**) CD4^+^ and CD8^+^ T cells were quantified by IHC using 10 randomly selected fields per section at 200× magnification, and the average count was used for comparison (n = 7 mice/group). (**G**) Total RNA was extracted from tumors of *WT* or *Cxcr2*^−/−^ mice at 4 weeks, and gene expression levels were evaluated by RT-qPCR (n = 6–15 mice/group). (**H**) CT26 cells (1 × 10^5^) were injected intravenously into *WT* and *Cxcr2*^−/−^ mice. At 3 weeks after inoculation, lungs were harvested and fixed in Bouin’s solution for quantification of metastatic nodules (n = 9 mice/group). Representative lung images (left), metastatic nodule counts (middle), and H&E sections (right; scale bar = 2 mm) are shown. Data are presented as mean ± SEM. (**A**,**C**(right),**E**,**F**,**H**) Unpaired Student’s *t*-tests. (**C**(left)) Two-way ANOVA. (**B**) Comparisons between day 0 and each time point were performed using unpaired Student’s *t*-tests. No symbol indicates that the difference was not significant. (**G**) Comparisons between *WT* and *Cxcr2*^−/−^ groups for each gene were performed using unpaired Student’s *t*-tests. No symbol indicates that the difference was not significant. * *p* < 0.05, ** *p* < 0.01, *** *p* < 0.001, ns, not significant.

To determine whether our findings were specific to the 4T1 model, we employed another well-characterized BALB/c cancer model, the CT26 colon cancer model [36]. CT26 cells did not express detectable levels of *Cxcr2* mRNA (Figure 6A). Similar to the 4T1 model, *Cxcl1* and *Cxcl2* expression was upregulated in CT26 tumors with comparable kinetics (Figure 6B). Surprisingly, in contrast to the 4T1 model, primary tumor growth was significantly accelerated in *Cxcr2*^−/−^ mice (Figure 6C).

Primary tumors at 4 weeks were analyzed for leukocyte infiltration and gene expression. According to the IHC analysis, infiltration of Ly6G^+^ TANs into both necrotic (Figure 6D) and non-necrotic (Figure 6E) lesions of primary tumors was markedly reduced in *Cxcr2*^−/−^ mice. By contrast, comparable numbers of CD4^+^ and CD8^+^ T cells were observed in tumors from both *WT* and *Cxcr2*^−/−^ mice (Figure 6F and Figure S3). Notably, expression levels of *Il1b*, *Gzmb*, *Prf1*, *Foxp3* and *Tbx21* were decreased in primary tumors from *Cxcr2*^−/−^ mice (Figure 6G). Since CT26 cells rarely metastasize to the lung, we employed an experimental metastasis model by intravascularly injecting CT26 cells and evaluated their colonization in the lung. Consistent with the 4T1 model, the number of tumor nodules in the lung was significantly higher in *Cxcr2*^−/−^ mice compared to *WT* mice (Figure 6H). Collectively, these results indicate that CXCR2-mediated TAN infiltration suppresses tumor growth and lung metastasis in the CT26 model; thus, the anti-tumor effects of TANs are not limited to the 4T1 model but may extend to other cancer types.

### 2.8. Combined High CXCR2/CXCR1 Expression and Neutrophil Infiltration Predict an Improved Prognosis in Basal-Subtype BC

**Figure 7:**
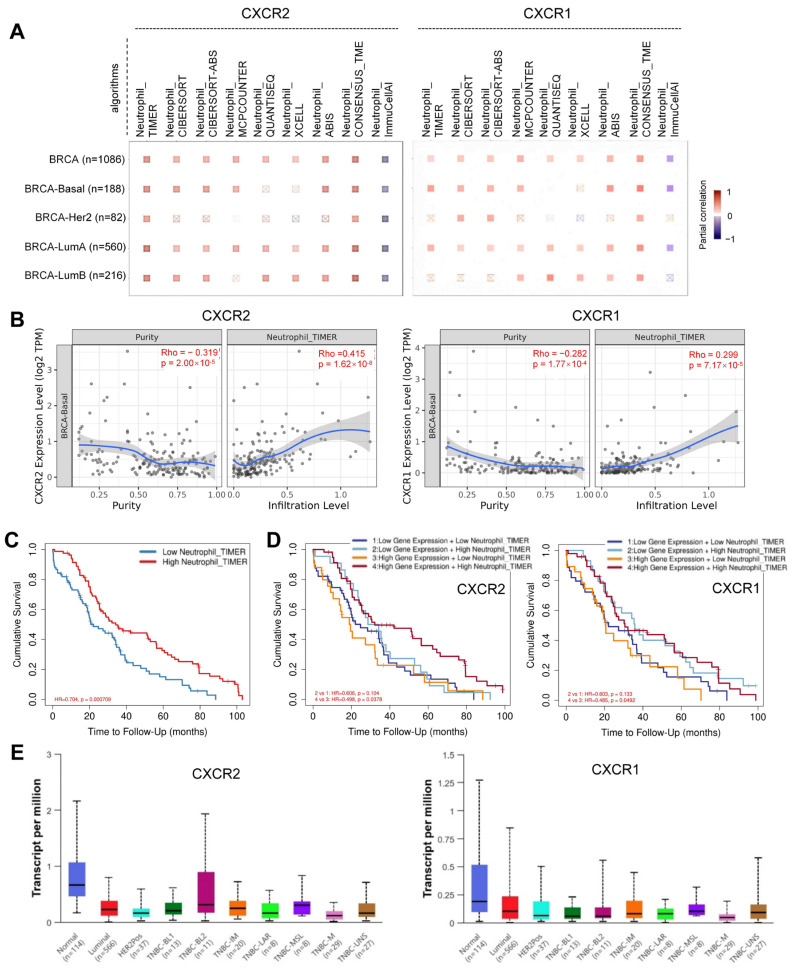
CXCR2 and CXCR1 expression correlate with neutrophil infiltration in BRCA basal-subtype patients. (**A**) Heatmap showing purity-corrected partial correlations between *CXCR2* and *CXCR1* expression and neutrophil infiltration across different breast cancer subtypes, as estimated by multiple algorithms (e.g., TIMER, CIBERSORT, MCPCOUNTER). Red squares indicate significant positive correlations (*p* < 0.05), whereas blue squares indicate negative correlations. (**B**) Spearman correlation analysis in the BRCA basal-like subtype using the TIMER algorithm. *CXCR2* (left) and *CXCR1* (right) expression levels were analyzed for their correlations with tumor purity and neutrophil infiltration. Crosses represent individual samples; the blue line indicates the fitted trend (regression curve), and the grey shaded area represents the 95% confidence interval. (**C**) Adjusted survival curves showing the association between neutrophil infiltration levels and overall survival (OS) over 100 months, based on a multivariable Cox proportional hazards model adjusting for age, pathological stage, and tumor purity. (**D**) Adjusted survival curves (truncated at 100 months) for patients stratified into four groups based on the median expression of *CXCR2* (left) or *CXCR1* (right) and the median level of neutrophil infiltration. Survival probabilities were estimated using a multivariable Cox proportional hazards model adjusted for age, pathological stage, and tumor purity. Differences in survival were compared between neutrophil-high and neutrophil-low groups within high and low receptor expression strata. (**E**) Expression levels of *CXCR2* (left) and *CXCR1* (right) in basal-type BRCA samples from the TCGA database. Box plots show mRNA expression in normal breast tissue and TNBC samples using the UALCAN platform. Data were derived from the TCGA-BRCA cohort filtered for the TNBC subtype. TNBC-BL1: TNBC Basal-like 1; TNBC-BL2: TNBC Basal-like 2; TNBC-IM: TNBC Immunomodulatory; TNBC-M: TNBC Mesenchymal; TNBC-MSL: TNBC Mesenchymal stem-like; TNBC-LAR: TNBC Luminal androgen receptor; TNBC-UNS: TNBC Unspecified.

To assess the clinical relevance of our findings from mouse models, we analyzed human transcriptomic data from The Cancer Genome Atlas (TCGA) using the TIMER3.0 platform [37]. Correlation analysis in the BC cohort was performed to evaluate the relationship between *CXCR2*/*CXCR1* expression and neutrophil infiltration. A heatmap of purity-adjusted partial correlations across BC subtypes revealed that, notably in the basal subtype (BRCA-Basal), *CXCR2*/*CXCR1* expression showed a consistent and significant positive association with neutrophil abundance across multiple algorithms (e.g., TIMER, CIBERSORT, MCPCOUNTER) (Figure 7A), indicating a pronounced association between *CXCR2*/*CXCR1* expression and neutrophil recruitment in this subtype.

As the basal-like molecular subtype, as defined by this platform, comprises approximately 70–80% of clinically classified TNBC [38], we further examined the relationship between *CXCR2* expression, tumor purity, and neutrophil infiltration within this subtype. Spearman correlation analysis (TIMER algorithm) demonstrated a negative correlation between *CXCR2* expression and tumor purity (Rho = −0.319, *p* = 0.00002), suggesting predominant expression within the TME rather than in tumor cells. *CXCR2* expression was positively correlated with neutrophil infiltration (Rho = 0.415, *p* = 0.0000000162) in basal-like BC (Figure 7B, left panel), indicating that elevated *CXCR2* levels are associated with increased neutrophil presence. Similarly, *CXCR1* expression negatively correlated with tumor purity (Rho = −0.282, *p* = 0.000177) and positively correlated with neutrophil infiltration (Rho = 0.299, *p* = 0.0000717) in basal-like BC (Figure 7B, right panel), supporting an immune microenvironment-derived expression pattern and its association with neutrophil abundance.

We next evaluated the prognostic impact of neutrophil infiltration in patients with basal-like BC. An initial unadjusted Kaplan–Meier analysis revealed that high neutrophil infiltration was significantly associated with improved overall survival (OS) (HR = 0.771, *p* = 0.00452) (Figure S4A). To determine whether this association was independent of potential confounders, we performed multivariable Cox proportional hazards analysis using the TIMER 3.0 platform, adjusting for patient age, pathological stage, and tumor purity (Figure S4B). Consistently, the high-infiltration group retained a significantly better OS compared with the low-infiltration group after adjustment (HR = 0.704, *p* = 0.000709) (Figure 7C). Although the significance of neutrophil abundance as a continuous variable was borderline in the multivariable Cox model (HR = 0.433, *p* = 0.059; Figure S4B), it showed a consistent trend toward a favorable prognosis in basal-like BC.

We then investigated whether this prognostic effect was modulated by median *CXCR2* or *CXCR1* expression. Initial joint analysis indicated that the survival benefit of neutrophils was more pronounced in the high-receptor subgroups (Figure S4C). To further assess this relationship while accounting for potential confounders, we applied the same multivariable-adjusted framework (Figure S4D,E). In the CXCR2-stratified analysis (Figure 7D, left panel), a survival benefit associated with high neutrophil infiltration was only observed in the high-*CXCR2* subgroup (Group 4 vs. 3, HR = 0.498, *p* = 0.038), but not in the low-*CXCR2* subgroup (Group 2 vs. 1, *p* = 0.104). This association remained significant after multivariable adjustment, supporting the functional importance of the CXCR2–neutrophil axis. Similarly, in the CXCR1-stratified analysis (Figure 7D, right panel), high neutrophil infiltration was associated with improved OS in the high-*CXCR1* subgroup (Group 4 vs. 3, HR = 0.485, *p* = 0.0492), whereas no significant difference was observed in the low-*CXCR1* subgroup (Group 2 vs. 1, *p* = 0.133). This trend was also maintained after adjustment. However, *CXCR1* expression was low in normal breast tissue (~0.2 TPM) and nearly undetectable across BRCA subtypes, particularly in TNBC (Figure 7E). Given its minimal expression, *CXCR1* appears less functionally relevant than *CXCR2*. Collectively, these data support our experimental findings and suggest that the CXCR2–neutrophil axis is a key contributor to favorable prognoses in basal-like BC and in TNBC.

## 3. Discussion

Neutrophils are the most abundant myeloid cells in circulation and exhibit pronounced functional plasticity within the TME [1,31]. Although traditionally viewed as innate effectors for microbial defense, recent evidence underscores their capacity to interface with tumor immunity. Depending on phenotypic cues and local context, neutrophils can either promote anti-tumor responses or facilitate tumor progression. Indeed, subsets of TANs exhibit antigen-presenting capacities and can enhance T-cell activation, while others adopt immunosuppressive phenotypes and contribute to tumor immune evasion [39]. In the present study, we used three mouse models deficient in FPR1, FPR2 or CXCR2 to demonstrate that neutrophil infiltration into 4T1 tumors, both primary and metastatic, is almost completely dependent on the interaction of CXCR2 on neutrophils with its ligands produced in the TME. A lack of neutrophil infiltration in *Cxcr2*^−/−^ mice resulted in increased lung metastasis in the 4T1 TNBC model and the CT26 colon cancer model and increased primary tumor growth in the CT26 model. Analysis of transcriptomic data from the human basal subtype of BC—accounting for approximately 70–80% of TNBCs [38]—revealed that high neutrophil infiltration, as well as high *CXCR2* and/or *CXCR1* expression, is associated with improved patient prognoses. These findings suggest that neutrophils infiltrating TNBC may exert an anti-tumor effect, in addition to their well-known pro-tumor activity.

Trafficking of neutrophils into injured tissues is regulated by multiple chemoattractant receptors, including classical chemoattractant receptors and chemokine receptors [5]. Although *Cxcr2*^−/−^ mice have been used to study the role of TANs in BC development and progression, it remained unclear whether other neutrophil chemoattractant receptors, such as FPR1 and FPR2, play a role in the recruitment of TANs. Two weeks after the inoculation of 4T1 cells, there were comparable levels of neutrophil infiltration in the necrotic and non-necrotic lesions of primary tumors in *WT*, *Fpr1*^−/−^ and *Fpr2*^−/−^ mice; however, only a few neutrophils were detected in *Cxcr2*^−/−^ mice. The absence of TANs in *Cxcr2*^−/−^ mice became apparent as early as 1 week after the inoculation. The profound loss of TANs in *Cxcr2*^−/−^ mice, affecting both the primary and the metastatic sites, justified using *Cxcr2*^−/−^ mice to study the role of TANs in the evolving tumor landscape.

To determine how the loss of TANs in the TME leads to increased lung metastasis, we examined both primary and metastatic tumors. Although there were no significant changes in angiogenesis or T cell recruitment in primary tumors, there were changes in the immune landscape. The expression of cytokines, such as *Il1b*, *Tnfa* and *Ifng*, was downregulated in primary tumors of *Cxcr2*^−/−^ mice. Notably, the expression of the cytotoxic molecules *Gzmb* and *Prf1,* which are expressed by CD8^+^ T cells, was significantly reduced from the initial stage of tumor development to the advanced stage; however, these multi-gene findings warrant cautious interpretation. This persistent reduction suggests that TANs may be essential for promoting the cytotoxic efficacy of CD8^+^ T cells throughout tumor development and progression stages. This immunological change was not restricted to the primary lesion as we also observed a similar attenuation of *Gzmb* and *Prf1* expression in metastatic lungs. In vitro, co-culture with *WT* neutrophils significantly augmented the expression of *Gzmb* and *Prf1* and tumor-killing capacity of CD8^+^ T cells. Thus, the absence of TANs in both primary and metastatic sites consistently blunts their functional output, which is particularly pronounced in the CD8^+^ compartment. A previous report showed that TANs can enhance T cell proliferation and IFN-γ production, often in a contact-dependent manner, thereby amplifying anti-tumor immunity against lung cancer [40]. We explored the possible involvement of ICAM-1 expressed on neutrophils in the interaction between neutrophils and CD8^+^ T cells. ICAM-1 is essential for the formation of stable immune synapses and effective TCR triggering [41]. However, blocking ICAM-1 only partially reduced the frequencies of GZMB^+^ CD8^+^ T cells following co-culture with neutrophils, indicating the presence of ICAM-1-independent mechanisms. In addition to ICAM-1, neutrophils can promote CD8^+^ T cell activation through costimulatory molecules, such as CD80 and CD86 [42]. Further studies are needed to elucidate the precise mechanisms through which neutrophils promote CD8^+^ T cell activation.

Several laboratories have investigated the involvement of CXCR2, which is expressed by both cancer cells and non-cancer cells, in TNBC progression using preclinical animal models. Knockdown of CXCR2 in 4T1 and CI66 cells, a less aggressive TNBC cell line, did not affect transplanted cell growth but significantly reduced spontaneous lung metastasis of CI66 cells [43]. There was no information as to the spontaneous lung metastasis of CXCR2-knockdown 4T1 cells. We examined *Cxcr2* expression in 4T1 cells and did not detect significant expression, which excludes the possibility of CXCR2-CXCR2 ligand interactions on cancer cells in our study. In another study, 4T1 and CI66 cells were orthotopically injected into *Cxcr2*^−/−^ mice [16]. The growth of the aggressive 4T1 tumors was not altered; however, the growth of the less aggressive CI66 tumors was significantly reduced in the absence of host CXCR2. Contrary to our results, the spontaneous lung metastasis of both 4T1 and CI66 cells was significantly reduced, suggesting a protumor activity of TANs in the models. A major difference between our approach and that of the other study is that primary tumors were resected when they reached the size of 0.5 cm^3^ and lung metastasis was evaluated 5 weeks after cancer cell inoculation. It is not clear how these technical differences influenced the overall outcomes. A previous study has shown that the stress of operative removal of 4T1 tumors leads to an increased number of MDSCs that preferentially infiltrate the TME and promote tumor metastasis [44]. Considering that many cancer patients undergo the resection of primary tumors, it is important to determine whether this treatment was responsible for the different outcomes between the two studies. Our findings are consistent with those of a previous study using the MMTV-PyMT mouse model of TNBC, which demonstrated increased lung metastasis in the absence of CXCR2 [18,19]. In contrast to the CI66 tumor model noted above, CT26 tumor growth was accelerated in *Cxcr2*^−/−^ mice. CT26 cells are more antigenic than 4T1 cells and responsive to immunotherapy [28], suggesting that CT26 tumors are under immune pressure. Therefore, when immune responses are reduced in the absence of TANs, which promote the activation of CD8^+^ T cells, CT26 tumor growth can be accelerated.

*Cxcr2*^−/−^ mice are known to exhibit an expansion of circulating neutrophils, with an increased proportion of immature phenotypes [15,45]. These immature neutrophils are frequently associated with immunosuppressive or dysfunctional states and share features with polymorphonuclear myeloid-derived suppressor cells (PMN-MDSCs) [46]. Consistent with this notion, Ly6G^+^ neutrophils from tumor-bearing *Cxcr2*^−/−^ mice failed to enhance CD8^+^ T cells activation induced by anti-CD3 and anti-CD28 antibodies in vitro, suggesting that the immature dysfunctional neutrophils in *Cxcr2*^−/−^ could contribute to the increased 4T1 cell metastasis. However, infiltration of neutrophils, including these dysfunctional neutrophils, was mostly absent in tumors of *Cxcr2*^−/−^ mice, suggesting minimal effects from the dysfunctional neutrophils. In *Cxcr2*^−/−^ mice, neutrophil infiltration was inhibited from the early stage after cancer cell inoculation, which may influence responses in later stages. To complement our study using systemic *Cxcr2*^−/−^ mice, we also depleted neutrophils in *WT* mice by intraperitoneally injecting anti-Ly6G antibody after primary tumor establishment and detected increased lung metastasis as well. Therefore, it is unlikely that the loss of early neutrophil infiltration in *Cxcr2*^−/−^ mice greatly contributed to the increased lung metastasis of 4T1 cells in the late stage.

Neutrophils can interact with circulating tumor cells and promote metastasis [47]. Previous work has shown that *Cxcr2*^−/−^ mice exhibit systemic alterations in neutrophil distribution, with increased neutrophil numbers in the bone marrow and spleen, enrichment of mature neutrophils in the bone marrow, and a higher proportion of immature neutrophils in the spleen [48]. Despite these changes, bone marrow neutrophils largely retain effector functions, whereas splenic neutrophils display attenuated activity, suggesting that CXCR2 deficiency primarily affects neutrophil maturation, distribution, and trafficking rather than intrinsic function. Consistent with this, we observed an increased proportion of circulating neutrophils in *Cxcr2*^−/−^ mice, with a higher percentage of immature CD101^−^Ly6G^+^ cells compared with *WT* mice (Figure S5A). In the lungs of tumor-bearing mice, however, the proportions of neutrophils (including mature and immature subsets), T cells, and macrophages were comparable between *WT* and *Cxcr2*^−/−^ mice (Figure S5B). These results suggest that the observed phenotypes in *Cxcr2*^−/−^ mice are unlikely to be driven by broad systemic immune alterations or major changes in baseline lung immune composition. Nevertheless, the use of systemic *Cxcr2*^−/−^ mice represents a limitation of this study. Future studies employing cell type-specific conditional deletion models will be required to more precisely define the neutrophil-intrinsic role of CXCR2 in tumor metastasis.

A previous study focusing on MDSCs reported that higher levels of MDSCs correlated with a poor prognosis in BC patients [49]. Another study analyzed scRNA-seq datasets in a public database and found that TNBC cases with high fractions of M2 macrophages and neutrophils were significantly associated with shorter survival times than cases with low fractions [50]. In the present study, we analyzed TCGA transcriptomic data and found that high neutrophil infiltration with high *CXCR2* or *CXCR1* expression was associated with a better prognosis in basal subtype BC. Similar results were obtained by others when neutrophil infiltration and the CXCR2 protein expression in human TNBC cases were examined by IHC. Although the level of neutrophil infiltration was not associated with a better prognosis, high CXCR2 staining levels were associated with a favorable prognosis [13,51]. Thus, our results support the previous IHC findings and suggest that neutrophil infiltration and the expression of *CXCR2* and/or *CXCR1* may serve as prognostic biomarkers in patients with TNBC, especially the basal subtype of BC. However, these findings are based on retrospective analyses of public transcriptomic datasets and online platforms and are therefore inherently limited by their correlative nature. To translate these prognostic biomarkers into clinical practice, further validation in large, multicenter cohorts and standardization of clinical IHC protocols for assessing the CXCR2–neutrophil axis are required.

Neutrophils are a highly heterogeneous cell population [1]. Single-cell RNA sequencing of human and murine datasets identified neutrophil transcriptomic subtypes and developmental lineages associated with health and malignancy. In PyMT-BC tumors, neutrophils were separated into healthy and tumor-specific subtypes. Within tumors, tumor-specific neutrophils were further separated into two subgroups: activated neutrophils which are transcriptionally similar to neutrophils from healthy tissue, and a tumor-specific subtype. These subtypes were also present in non-small cell lung cancer and colon cancer. Further analysis of transcripts expressed in colon cancer liver metastases revealed the presence of an additional transcriptionally segregated neutrophil population specific to metastatic cancer, called the metastasis-specific population. Marker genes for this population included *TXNIP*, *RIPOR2/FAM65B* and *STK17B*, which are important for late T-cell expansion, naive T-cell quiescence, survival, and activation, suggesting they are T cell-suppressive. Interestingly, this cell population was also enriched for *CXCR2* expression, strongly suggesting that *CXCR2*-positive cells are functionally immune-suppressive [52]. Since many metastatic nodules are found in lungs of 4T1 tumor-bearing *WT* mice, a pro-tumor metastasis-specific TAN population is likely present with a healthy anti-tumor TAN population. In our study, almost all TANs, including both anti-tumor and pro-tumor TANs, were missing in both primary and metastatic tumors in *Cxcr2*^−/−^ mice and lung metastases were increased. This suggests that a significant magnitude of neutrophil-mediated anti-tumor activity is present in the lung of 4T1 tumor-bearing *WT* mice. The specific TAN subsets involved in regulating metastasis in our study remain to be defined. In particular, single-cell RNA sequencing would be helpful to identify different TAN populations infiltrating tumors of *WT* and *Cxcr2*^−/−^ mice.

In conclusion, our study suggests that CXCR2-dependent TAN infiltration is a critical component of anti-tumor immunity in TNBC. Loss of TAN infiltration disrupts neutrophil–CD8^+^ T cell interactions required for CD8^+^ T cell activation and impairs direct tumor cell killing, thereby promoting lung metastasis despite preserved CD8^+^ T cell infiltration. These findings highlight the functional complexity of TANs in cancer and suggest that the prognostic significance of CXCR2 signaling and neutrophil trafficking should not be considered uniformly pro-tumorigenic across different contexts. Strategies that integrate the anti-tumor functions of neutrophils with CD8^+^ T cell activity may provide greater prognostic insight and clinical relevance for the management of TNBC, particularly basal-like BC.

## 4. Materials and Methods

### 4.1. Mice

BALB/c mice were purchased from Japan SLC, Inc. (Hamamatsu, Japan). *Fpr1*^−/−^ and *Fpr2*^−/−^ mice, originally generated on a C57BL/c background [31,53,54], were backcrossed onto a BALB/c background for 10 generations in Dr. Ji Ming Wang’s laboratory (National Cancer Institute, National Institute of Health, Frederick, MD, USA). *Cxcr2*^−/−^ mice on a BALB/c background were purchased from the Jackson Laboratory (Bar Harbor, ME, USA). All 3 mouse strains were obtained from Dr. Ji Ming Wang. Mice were bred and maintained under specific pathogen-free conditions on a 12-h light/dark cycle at an ambient temperature of 21 °C, with ad libitum access to food and water, at the Department of Animal Resources, Okayama University (Okayama, Japan). Mice at 8 weeks of age were used in this study. To minimize potential confounders, mice were housed under identical environmental conditions. Treatments and measurements were performed using standardized procedures across groups.

### 4.2. Cell Lines

4T1 and CT26 cells were obtained from the American Type Culture Collection (ATCC, Manassas, VA, USA). Cells were cultured in RPMI-1640 medium (Sigma-Aldrich, St. Louis, MO, USA), supplemented with 10% fetal bovine serum (FBS, Gibco, Carlsbad, CA, USA), 100 U/mL penicillin and 100 ug/mL streptomycin (Sigma-Aldrich, St. Louis, MO, USA) at 37 °C in a humidified incubator with 5% CO_2_.

### 4.3. Tumor Models

For the 4T1 model, female mice were inoculated with 4T1 cells (1 × 10^5^ cells in 100 μL of Phosphate-Buffered Saline (PBS)) via mammary fat pad injection. For the CT26 model, male mice were challenged with CT26 (1 × 10^5^ cells in 100 μL of PBS) by subcutaneous injection into the dorsal flank. Body weight and tumor size were measured 3 times per week using a caliper, and tumor volume (length × width^2^ × 0.52) was calculated. Mice were euthanized when tumors reached 20 mm in diameter or when signs of distress were observed. For the experimental metastasis model, male mice were intravenously injected with CT26 cells (1 × 10^5^ cells in 100 μL PBS). At indicated time intervals, the mice were anesthetized, bled, euthanized, and the tumors and lungs were collected. Prior to lung harvest, mice were euthanized and transcardially perfused with heparinized PBS (5–10 U/mL) via the right ventricle until the lungs were visibly cleared of blood. Then, the lungs were processed for flow cytometry or immunohistochemistry, as described below. To deplete neutrophils, female WT mice were randomly assigned to two groups. On days 5, 8, and 11 after tumor inoculation, the mice were administered either an anti-Ly6G monoclonal antibody (100 μg in 100 μL of PBS, clone 1A8; Bio X Cell, Lebanon, NH, USA) or an isotype control antibody (100 μg in 100 μL of PBS) intraperitoneally. Depletion efficiency was verified using IHC on tissue samples. The experimental unit was an individual mouse. The primary outcome measure was tumor growth (tumor volume). No formal a priori sample size calculation was performed; sample size was determined based on previous studies using the same model and our prior experience. Mice were randomly assigned to experimental groups. No animals or data points were excluded from the analysis. No predefined exclusion criteria were established. No unexpected adverse events were observed during the study. Investigators were aware of group allocation throughout the study, including during allocation, conduct of the experiments, outcome assessment, and data analysis.

All animal procedures were approved by the Animal Care and Use Committee of Okayama University and were performed in accordance with institutional guidelines. A formal study protocol was prepared prior to the initiation of the experiments; however, it was not publicly registered.

### 4.4. Immunohistochemistry (IHC)

Tissue specimens were fixed in 10% formalin and embedded in paraffin. Sections (4 μm-thick) were deparaffinized and rehydrated through a graded ethanol series. Endogenous peroxidase activity was blocked by 3% hydrogen peroxide for 10 min at room temperature. For antigen retrieval, sections were immersed in 10 mM citrate buffer (pH 6.0) or 5 mM EDTA solution (pH 8.0) and heated in a pressure cooker (700 W) for 25 min. After cooling to room temperature, sections were incubated with protein blocking buffer (Dako, Santa Clara, CA, USA), followed by incubation with primary antibodies at 4 °C for 1.5 h. Immunostaining was performed using the indirect avidin-biotin-peroxidase method with appropriate secondary antibodies for 30 min at room temperature and visualized with DAB (Dako) according to the manufacturer’s instructions. Sections were counterstained with hematoxylin. Images were acquired using a DP73 digital camera (Olympus, Tokyo, Japan) under a microscope. For immune cell quantification, ten non-overlapping high-power fields (HPFs) were randomly selected per tumor section, excluding necrotic regions. F4/80^+^, CD8^+^, CD4^+^, and ERG^+^ cells that were positively stained with clearly identifiable nuclei and specific chromogenic signals were manually counted in each field. The number of positive cells per HPF was recorded, and the mean across fields was used for statistical analysis. For analysis of immune cell infiltration in lung metastatic lesions, Ly6G^+^, CD4^+^, and CD8^+^ cells were quantified within metastatic foci. As metastatic lesions varied in size between groups, cell counts were normalized to lesion area. The number of positive cells was counted, and lesion areas were measured using ImageJ software (Version 1.53, National Institutes of Health, Bethesda, MD, USA). Immune cell density was expressed as cells per unit area (cells/mm^2^). For CD31 staining, microvessel density was assessed using the hotspot method. Briefly, areas with the highest vascular density were identified at low magnification, and individual CD31^+^ vascular structures with a visible lumen or endothelial cell cluster were counted as single microvessels in each hotspot HPF. Ten hotspot HPFs were analyzed per sample, and the mean value was used for statistical comparison. All samples were evaluated using the same criteria to minimize observer bias. Antibodies for immunohistochemistry used in this study are shown in Table S1.

### 4.5. Isolation of Single Cells from Immune Organs and Tumors of Tumor-Bearing Mice

Single-cell suspensions from lungs and spleens were prepared by mechanical dissociation followed by filtering through a 70 μm cell strainer. Cells were passed through nylon mesh, subjected to red blood cell lysis, and resuspended in flow cytometry buffer. For tumor tissues, tumors were excised, minced into small pieces, and digested with type-IV collagenase, hyaluronidase, and DNase I (0.05 mg/mL each) for 30 min at 37 °C. Tissues were further dissociated using a gentleMACS Dissociator (Miltenyi Biotec, Bergisch Gladbach, Germany) and filtered through a 70 μm cell strainer (Corning, Corning, NY, USA) to obtain single-cell suspensions. Red blood cells were removed using red blood cell lysis buffer.

### 4.6. Isolation of Neutrophils from Tumor-Bearing Mice

Tumor-bearing mice were anesthetized with isoflurane and whole blood was collected by terminal cardiac puncture into EDTA-coated syringes. After red blood cell lysis, leukocytes were resuspended in PBS containing 0.05% Bovine Serum Albumin (BSA). Neutrophils were isolated by magnetic-activated cell sorting (MACS) using anti-Ly6G microbeads (Miltenyi Biotec) according to the manufacturer’s instructions. Ly6G^+^ cells were enriched using MS columns (Miltenyi Biotec) in a magnetic field, filtered through a 40 μm cell strainer, and resuspended in PBS containing 0.05% BSA, and used immediately. Purity routinely exceeded 90% Ly6G^+^CD11b^+^, as assessed by flow cytometry.

### 4.7. Isolation of CD8^+^ T Cells

CD8^+^ T cells were isolated from mouse spleens by negative selection using a CD8a^+^ T Cell Isolation Kit (Miltenyi Biotec) according to the manufacturer’s instructions. Briefly, splenocytes were prepared by mechanical dissociation followed by red blood cell lysis. Cells were incubated with a cocktail of biotin-conjugated antibodies and anti-biotin magnetic microbeads, and CD8^+^ T cells were obtained by magnetic separation using an LS column (Miltenyi Biotec). Purity was approximately 87% as assessed by flow cytometry.

### 4.8. In Vitro Co-Culture of CD8^+^ T Cells with Neutrophils

CD8^+^ T cells were resuspended in complete RPMI-1640 (10% FBS, 1% penicillin/streptomycin, 50 µM 2-mercaptoethanol) and plated at 1 × 10^6^ cells per well in 24-well plates. Neutrophils were added at the indicated effector-to-target ratios of 1:1. T cells were stimulated with plate-bound anti-CD3 (3 µg/mL) and soluble anti-CD28 (3 µg/mL) and cultured for 48 h at 37 °C in 5% CO_2_. Intracellular granzyme B (GZMB) and perforin 1 (PRF1) expression was assessed by flow cytometry. Stimulated CD8^+^ T cells cultured without neutrophils served as controls. In a separate experiment, under neutrophil and CD8^+^ T cell co-culture conditions (1:1 ratio), an anti-ICAM-1 monoclonal antibody (Thermo Fisher, clone YN1/1.7.4, Waltham, MA, USA) or an isotype-matched Rat IgG2b control was added to the culture medium at a final concentration of 1 μg/mL to block ICAM-1 signaling.

For transwell assays, CD8^+^ T cells (1 × 10^6^ cells) were placed in the lower chamber of a 24-well plate in 600 µL of complete medium, and neutrophils (1 × 10^6^ or 2 × 10^6^ cells) were seeded in a 0.4 µm pore-size transwell insert (Corning, Corning, NY, USA) in 200 µL of medium. T cells were stimulated as described above and cultured for 48 h at 37 °C in 5% CO_2_. GZMB and PRF1 expression was analyzed as in the direct co-culture experiment.

### 4.9. Tumor Cell-Killing Assays

Tumor cell cytotoxicity was assessed using CFSE-labeled 4T1 cells. Briefly, 4T1 cells were labeled with 5 µM CFSE (Invitrogen, Carlsbad, CA, USA; Cat. No. C34554) in PBS for 10 min at 37 °C, quenched with complete medium, washed twice, and seeded in 24-well flat-bottom plates (5 × 10^4^ cells per well). Cells were allowed to adhere overnight. To assess the anti-tumor efficacy of neutrophils, freshly isolated neutrophils were directly added to CFSE-labeled 4T1 cells at the indicated E:T ratios (typically 10:1) and co-cultured for 48 h. To evaluate the effect of neutrophils on CD8^+^ T cell-mediated anti-tumor activity, CD8^+^ T cells were first co-cultured with neutrophils in complete RPMI-1640 for 48 h, as described above. CD8^+^ T cells were then re-isolated by negative selection using a CD8a^+^ T Cell Isolation Kit (Miltenyi Biotec) and added to CFSE-labeled 4T1 cells at an effector-to-target ratio of 10:1. After 48 h of co-culture, both adherent and non-adherent cells were collected. At the end of incubation, cells were stained with DAPI. Cytotoxicity was quantified by flow cytometry as the percentage of CFSE^+^DAPI^+^ cells out of the total CFSE^+^ tumor cells and normalized to the spontaneous death rate in target-only controls.

### 4.10. Reverse Transcription Quantitative PCR (RT-qPCR)

Tissues were homogenized in Trizol Reagent (Thermo Fisher Scientific, Waltham, MA, USA) and total RNA was isolated using High Pure RNA Isolation Kit (Roche Applied Science, Mannheim, Germany). First-strand cDNA was synthesized from 2 μg of total RNA using the High-capacity cDNA Reverse Transcription Kit (Thermo Fisher Scientific, Waltham, MA, USA). RT-qPCR was run using a StepOnePlus system (Thermo Fisher Scientific). Gene expression levels were normalized to *Gapdh* as the internal control. TaqMan Gene Expression Assays used in this study are listed in Table S2.

### 4.11. Western Blotting

Tissue proteins were extracted using RIPA lysis buffer (50 mM Tris-HCl, pH 7.4; 150 mM NaCl; 1% NP-40; 0.5% sodium deoxycholate; 0.1% SDS) supplemented with protease and phosphatase inhibitors. Protein samples (15–30 μg) were separated on 4–12% NuPAGE Bis-Tris precast gels (Thermo Fisher Scientific) and transferred onto nitrocellulose membranes (GE Healthcare Life Science, Chicago, IL, USA). Membranes were blocked with 5% skim milk and incubated overnight at 4 °C with primary antibodies, followed by incubation with horseradish peroxidase (HRP)-conjugated secondary antibodies for 1 h at room temperature. After washing, target proteins were detected using a C-DiGit Blot scanner (LI-COR Biosciences, Lincoln, NE, USA). Blot intensities were semi-quantitated using Image Studio software (Version 5.2, LI-COR Biosciences, Lincoln, NE, USA). Antibodies used for Western blotting are listed in Table S3.

### 4.12. Flow Cytometry

Single-cell suspensions were prepared from tissues as described above. Cells (1 × 10^6^ cells) were washed once with rinsing buffer, blocked using anti-CD16/32 (BioLegend, San Diego, CA, USA) to prevent non-specific binding, and stained with antibodies to cell surface markers. For intracellular staining, cells were fixed with fixation buffer and stained with antibodies diluted in permeabilization buffers (BioLegend). Data acquisition was performed using a CytoFLEX LX flow cytometer (Beckman Coulter, Brea, CA, USA), and analysis was conducted with CytExpert software (version 2.5). Antibodies used for flow cytometry are listed in Table S4.

### 4.13. ELISA

CXCL1 and CXCL2 levels in cell culture supernatants were measured using mouse ELISA kits (Thermo Fisher Scientific, Cat. No. EMKC L20 and EMCXCL2) according to the manufacturer’s instructions. Supernatants were centrifuged to remove debris and analyzed in duplicate. Absorbance was measured at 450 nm, and cytokine concentrations were determined from standard curves.

### 4.14. Database Analysis

To investigate the clinical significance and immune relevance of CXCR1 and CXCR2 in the basal-like BC (BRCA), we performed an integrated bioinformatics analysis across multiple independent clinical datasets. mRNA expression levels of CXCR1 and CXCR2 in basal-like BC tissues compared with normal mammary tissues were analyzed using UALCAN (http://ualcan.path.uab.edu) based on TCGA Breast Invasive Carcinoma (BRCA) datasets. To evaluate the independent prognostic value of tumor-associated neutrophils (TANs) and CXCR1/2, we used the TIMER 3.0 platform to analyze the TCGA-BRCA basal-like cohort (https://compbio.cn/timer3/ (accessed on 20 February 2026)). To control for potential confounding clinical variables, multivariable Cox proportional hazards regression models were constructed to adjust for patient age, pathological stage (I–IV), and tumor purity. TAN abundance was estimated using the TIMER algorithm. Survival analysis was restricted to a follow-up period of 100 months to ensure clinical relevance while minimizing bias from long-term loss to follow-up or non-cancer-specific mortality. All statistical parameters, including *p*-values, hazard ratios (HRs), and 95% confidence intervals (Cis), were obtained directly from the respective platforms to ensure methodological transparency and rigorous cross-database validation.

### 4.15. Statistical Analysis

Statistical analyses were performed using GraphPad Prism 9.0 (GraphPad Software, San Diego, CA, USA). Data distribution was assessed for normality using the Shapiro–Wilk test, and homogeneity of variances was evaluated using Brown-Forsythe or Bartlett’s tests, as appropriate. For comparisons between two groups with normally distributed data, a two-tailed unpaired Student’s *t*-test was used. Comparisons among three or more groups were performed using one-way analysis of variance (ANOVA) followed by Tukey’s multiple comparison test. Experiments involving two independent variables or longitudinal measurements (e.g., tumor growth curves) were analyzed using two-way ANOVA followed by Sidak’s multiple comparison test, with repeated measurements within the same subjects treated as matched data. Although non-parametric tests (Mann–Whitney and Kruskal–Wallis tests) were pre-specified for use when normality assumptions were not met, all datasets satisfied the criteria for parametric analysis. All experiments were performed with at least three independent biological replicates unless otherwise indicated. Data from independent experiments were pooled for statistical analysis as consistent trends were observed across replicates. Data are presented as mean ± SEM. A *p* value < 0.05 was considered statistically significant.

## Data Availability

The original contributions presented in this study are included in the article/Supplementary Materials. Further inquiries can be directed to the corresponding authors.

## Supplementary Materials

The following supporting information can be downloaded at: https://www.mdpi.com/article/10.3390/ijms27073143/s1.
