# Supplementary material for: CXCR2-Dependent Infiltration of Tumor-Associated Neutrophils Is Linked to Enhanced CD8+ T Cell Effector Function and Reduced Lung Metastasis in 4T1 Breast Cancer
Source: Int J Mol Sci. 2026 Mar 30;27(7):3143. doi: 10.3390/ijms27073143 (PMC13073189; doi:10.3390/ijms27073143)
Supplement: Supplementary file 1 [file ijms-27-03143-s001.zip › Revised Supplementary Figure.pdf]

Fig. S1

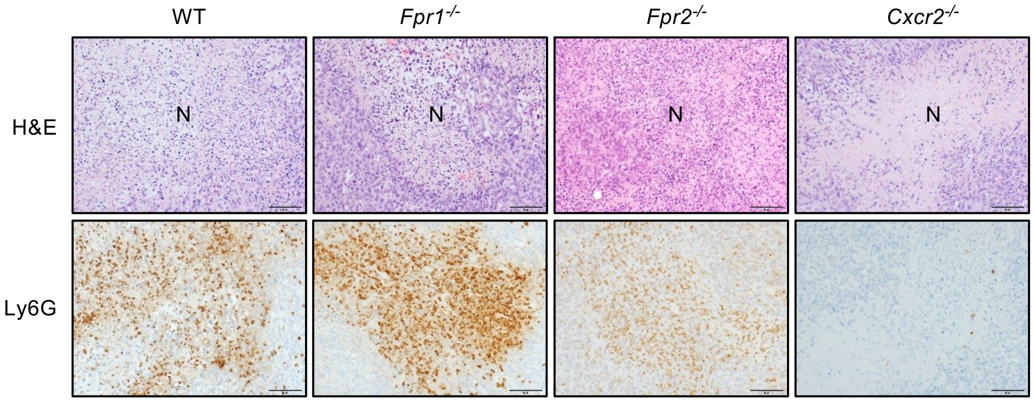

Figure S1. Neutrophil infiltration in 4T1 primary tumors from *WT*, *Fpr1*$^{-/-}$, *Fpr2*$^{-/-}$, and *Cxcr2*$^{-/-}$ mice. 4T1 cells (1×10^5) were inoculated into the mammary fat pad of female *WT*, *Fpr1*$^{-/-}$, *Fpr2*$^{-/-}$, and *Cxcr2*$^{-/-}$ mice. Representative H&E (upper panels) and anti-Ly6G immunohistochemical staining (lower panels) of tumor sections harvested at 2 weeks are shown. Scale bar = 100 μm. N indicates necrotic areas.

Fig. S2

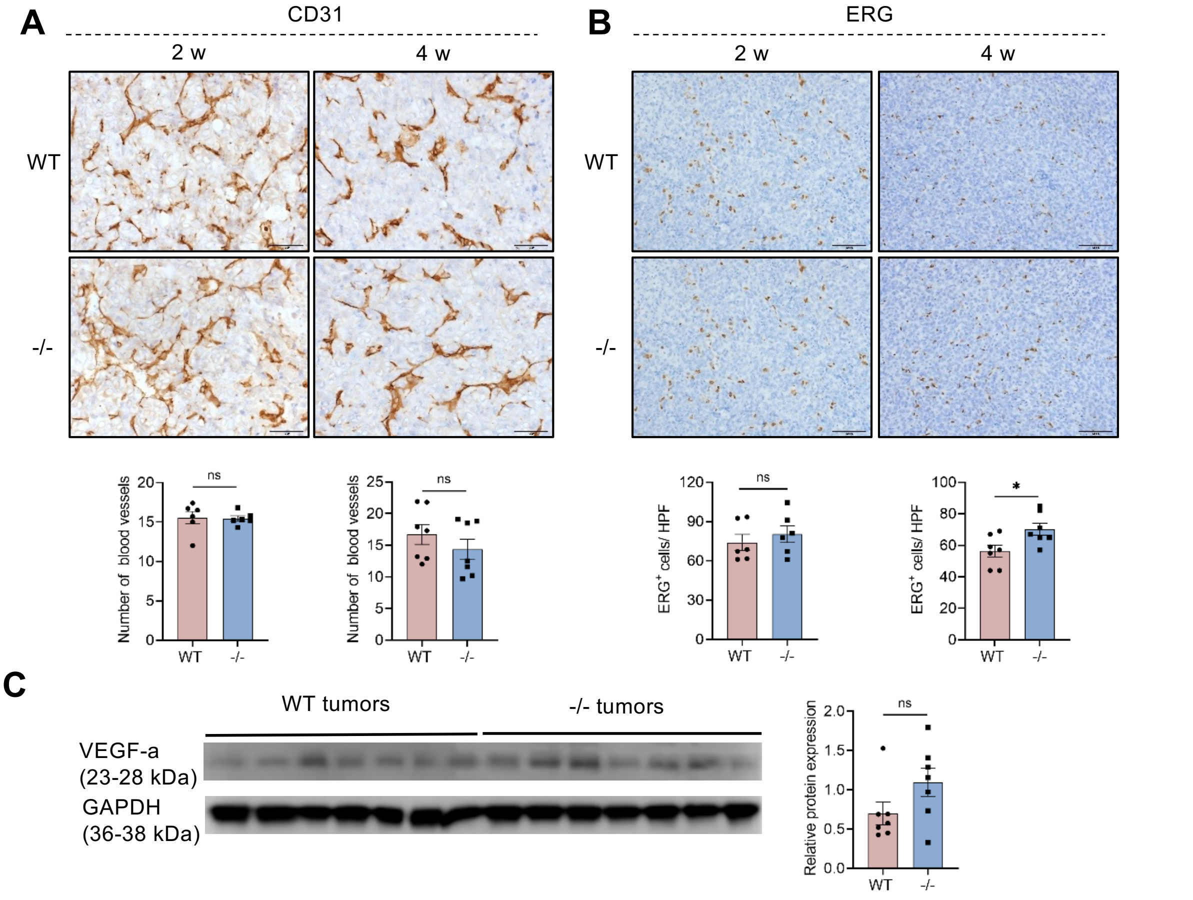

Figure S2. Angiogenesis in 4T1 primary tumors from *WT* and *Cxcr2*$^{-/-}$ mice. 4T1 cells (1 × 10^5) were inoculated into the mammary fat pad of female *WT* and *Cxcr2*$^{-/-}$ mice. (A, B) Primary tumor sections collected at 2 and 4 weeks were analyzed by immunohistochemistry using anti-CD31 (A) or anti-ERG (B) antibodies. Representative images are shown in the upper panels (scale bar = 50 μm for A; 100 μm for B). The lower panels show quantification of CD31$^+$ (A) or ERG$^+$ (B) blood vessels. Ten hot spot fields per section were randomly selected and analyzed at 400× (A) or 200× (B) magnification, and the average number of positive vessels was used for comparison (n = 6–7 mice per group). Data are presented as mean ± SEM. Student's t-test was used for comparisons. *$p < 0.05$, ns; not significant.

Fig. S3

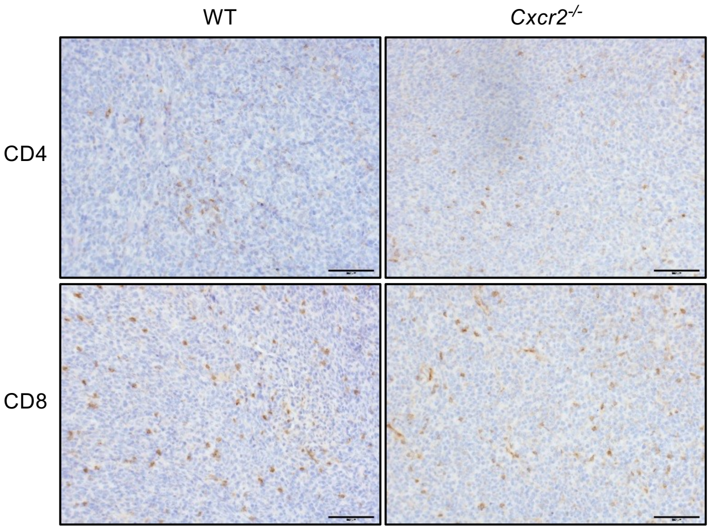

Figure S3. $CD4^+$ and $CD8^+$ T cell infiltration in CT26 primary tumors from *WT* and *Cxcr2*$^{-/-}$ mice. CT26 cells (1×10^5) were injected subcutaneously into the dorsal flank of *WT* and *Cxcr2*$^{-/-}$ mice. Mice were euthanized at 4 weeks, and primary tumors were harvested. Representative anti-CD4 and anti-CD8 immunohistochemical staining of tumor sections are shown. Scale bar = 100 μm.

Fig. S4

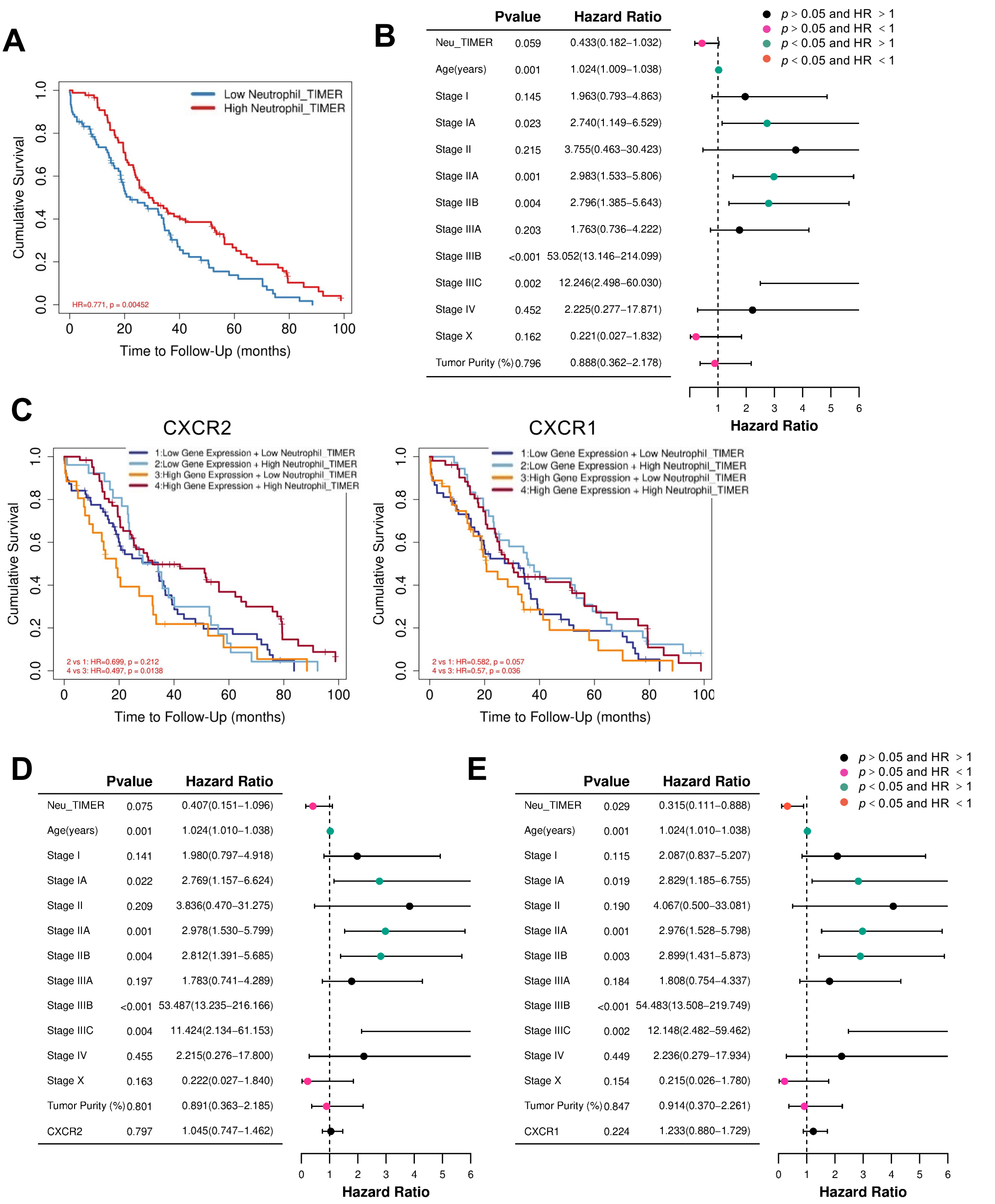

Figure S4. Univariate analysis and Multivariate Cox regression analysis of neutrophil infiltration and CXCR1/2 expression in Basal-like breast cancer. Multivariate Cox models were adjusted for age, pathological stage (I–IV), and tumor purity. Neutrophil infiltration was estimated using the TIMER algorithm. Hazard ratios (HRs) with 95% confidence intervals (CIs) are shown. (A) Kaplan–Meier curves of overall survival (OS) stratified by neutrophil infiltration (up to 100 months). (B) Multivariable analysis of neutrophil infiltration and clinical factors. (C) Kaplan–Meier curves stratified by median CXCR2 (left) or CXCR1 (right) expression and neutrophil infiltration. (D, E) Multivariable analyses evaluating the independent prognostic value of CXCR2 (D) or CXCR1 (E) with neutrophil infiltration.

Fig. S5

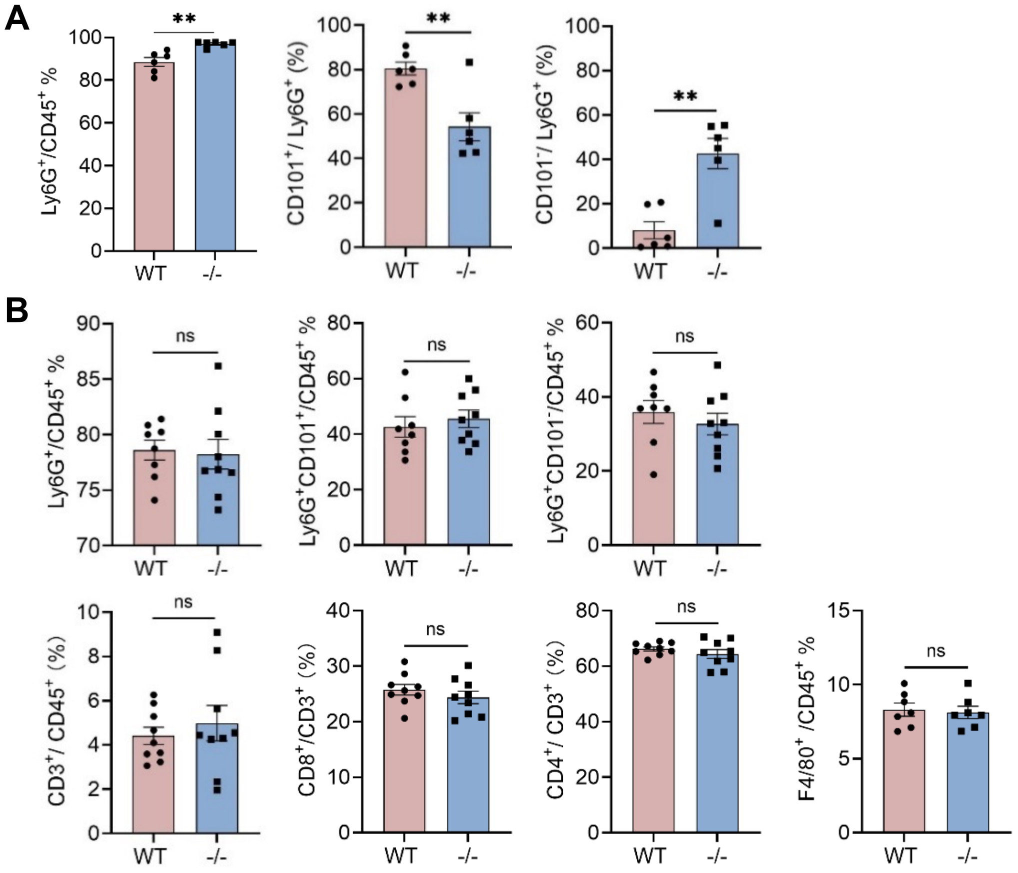

Figure S5. Comparisons of immune cell populations in *WT* or *Cxcr2*$^{-/-}$ mice. (**A**) Flow cytometric analysis of peripheral blood neutrophils from tumor-bearing *WT* or *Cxcr2*$^{-/-}$ mice (n = 6 mice/group). (B) Flow cytometric analysis of immune cell populations in the lungs of tumor-bearing *WT* or *Cxcr2*$^{-/-}$ mice (n = 7-9 mice/group). Data are presented as mean ± SEM. Student's t-test was used for comparisons. ***p* < 0.01, ns; not significant.
