# Supplementary material for: CXCR2-Dependent Infiltration of Tumor-Associated Neutrophils Is Linked to Enhanced CD8+ T Cell Effector Function and Reduced Lung Metastasis in 4T1 Breast Cancer
Source: Int J Mol Sci. 2026 Mar 30;27(7):3143. doi: 10.3390/ijms27073143 (PMC13073189; doi:10.3390/ijms27073143)
Supplement: Supplementary file 1 [file ijms-27-03143-s001.zip › Revised Supplementary tables.pdf]

Table S1. Antibodies used for immunohistochemistry in this study

Antibody	Company (Cat. number)	Dilution
Ly6G	BioLegend (127601)	1:100
F4/80(D2S9R)	Cell Signaling Technology (70076)	1:500
CD4(D7D3Z)	Cell Signaling Technology (25229)	1:2000
CD8α（D4W2Z）	Cell Signaling Technology (98941)	1:400
CD31	Cell Signaling Technology (97249)	1:200
ERG(A7L1G)	Abcam (Ab182981)	1:500
HRP-labeled Second antibody		
Histofine Simple Stain Mouse MAX-PO(Rabbit)	Nichirei Bioscience Inc.(414341)	
Histofine Simple Stain Mouse MAX-PO(Rat)	Nichirei Bioscience Inc.(414311)	

BioLegend: San Diego, CA, USA
Cell Signaling Technology: Danvers, MA, USA
Abcam: Cambridge, UK
Nichirei Bioscience Inc.: Tokyo, Japan

Table S2. Taqman gene expression assays used for RT-qPCR

Gene	Taqman gene expression assay kit
Cxcr2	Mm99999117_s1
Fpr1	Mm00442803_s1
Fpr2	Mm004844644_s1
Cxcl1	Mm04207460_m1
Cxcl2	Mm00436450_m1
GAPDH	Mm99999915_g1
Tnfa	Mm00443258_m1
Ifng	Mm01168134_m1
Nos2	Mm02525720_s1
Arg1	Mm00475988_m1
Gzmb	Mm00442837_m1
Prf1	Mm00812512_m1
Tbx21	Mm00450960_m1
Foxp3	Mm00475162_m1
Cxcl9	Mm00434946_m1
Cxcl10	Mm00445235_m1

Table S3. Antibodies used for Western blotting in this study

**Antigen**	**Company (Cat. number)**
Annexin A1	Proteintech (66344-1-1g)
VEGFa	Abcam (GR145343-2)
GAPDH	Cell Signaling Technology (2118s)
HRP-goat anti-rabbit IgG	Cell Signaling Technology (7074)
HRP-anti-mouse IgG	Cell Signaling Technology (7076)

Cell Signaling Technology: Danvers, MA, USA

Proteintech: Rosemont, IL, USA

Abcam: Cambridge, UK

Table S4. Antibodies used in flow cytometry in this study

Antigen	Company (Cat. number)
Ly6G (PE)	Biolegend (B473303)
CD45 (APC)	Biolegend (103112)
CD11b (APC)	Biolegend (101212)
CD3 (Pacific Blue)	Biolegend (100214)
CD8 (PE)	Biolegend (100706)
CD4 (FITC)	Biolegend (100406)
7AAD	Biolegend (420404)
F4/80 (FITC)	Biolegend (123108)
CD206 (APC)	Biolegend (141708)
iNOS (PE)	Biolegend (696806)
CD101 (APC)	Miltenyi Biotec (130-120-173)
Granzyme B (PE)	Biolegend (372207)
Zombie Aqua Fixable Viability	Biolegend (423101)
Ly6g (FITC)	Biolegend (B422492)
CD8a (APC-Cyanine7)	Biolegend (100714)
CD54/ICAM1 (PE)	Biolegend (116107)
CD45 (Pacific Blue)	Biolegend (103126)
CD11b (Percp-cy5.5)	Biolegend (101228)
CD45 (APC-Cyanine7)	Biolegend (103116)
Perforin (PE)	eBioscience (12-9392-80)
DAPI	NPE System (5735)
CFSE	Invitrogen (C3455)
Purified anti-mouse CD16/32	Biolegend (B401727)
Purified anti-mouse CD3	Biolegend (100202)
Purified anti-mouse CD28	Biolegend (102101)

Biolegend: San Diego, CA, USA

NPE system: Florida, USA

Miltenyi Biotec: Bergisch Gladbach, Germany

eBioscience: San Diego, CA, USA

Invitrogen, Thermo Fisher Scientific: Waltham, MA, USA
